# On the Choice and Number of Microarrays for Transcriptional Regulatory Network Inference

**DOI:** 10.1186/1471-2105-11-454

**Published:** 2010-09-09

**Authors:** Elissa J Cosgrove, Timothy S Gardner, Eric D Kolaczyk

**Affiliations:** 1 Department of Biomedical Engineering, Boston University, Boston, MA, USA; 2 Department of Mathematics and Statistics, Boston University, Boston, MA, USA; 3 Amgen Inc., South San Francisco, CA, USA; 4 Amyris Biotechnologies, Emeryville, CA, USA

## Abstract

**Background:**

Transcriptional regulatory network inference (TRNI) from large compendia of DNA microarrays has become a fundamental approach for discovering transcription factor (TF)-gene interactions at the genome-wide level. In correlation-based TRNI, network edges can in principle be evaluated using standard statistical tests. However, while such tests nominally assume independent microarray experiments, we expect dependency between the experiments in microarray compendia, due to both project-specific factors (e.g., microarray preparation, environmental effects) in the multi-project compendium setting and effective dependency induced by gene-gene correlations. Herein, we characterize the nature of dependency in an *Escherichia coli *microarray compendium and explore its consequences on the problem of determining which and how many arrays to use in correlation-based TRNI.

**Results:**

We present evidence of substantial effective dependency among microarrays in this compendium, and characterize that dependency with respect to experimental condition factors. We then introduce a measure *n*_*eff *_of the effective number of experiments in a compendium, and find that corresponding to the dependency observed in this particular compendium there is a huge reduction in effective sample size i.e., *n*_*eff *_= 14.7 versus *n *= 376. Furthermore, we found that the *n*_*eff *_of select subsets of experiments actually exceeded *n*_*eff *_of the full compendium, suggesting that the adage 'less is more' applies here. Consistent with this latter result, we observed improved performance in TRNI using subsets of the data compared to results using the full compendium. We identified experimental condition factors that trend with changes in TRNI performance and *n*_*eff *_, including growth phase and media type. Finally, using the set of known E. coli genetic regulatory interactions from RegulonDB, we demonstrated that false discovery rates (FDR) derived from *n*_*eff *_-adjusted p-values were well-matched to FDR based on the RegulonDB truth set.

**Conclusions:**

These results support utilization of *n*_*eff *_as a potent descriptor of microarray compendia. In addition, they highlight a straightforward correlation-based method for TRNI with demonstrated meaningful statistical testing for significant edges, readily applicable to compendia from any species, even when a truth set is not available. This work facilitates a more refined approach to construction and utilization of mRNA expression compendia in TRNI.

## Background

With the availability of genome-wide mRNA expression data from DNA microarray experiments, transcriptional regulatory network inference (TRNI) from large compendia of these microarrays has become a fundamental task in computational systems biology. In this approach, transcription factor (TF)-gene interactions are predicted based on observed trends in mRNA expression across many experimental conditions. Unsupervised pairwise methods for TRNI, including relevance networks [1,2], partial correlation methods [3,4], graphical Gaussian models (GGM) [5], and context likelihood of relatedness (CLR) [6], are attractive as they do not require prior knowledge of the network and have been successfully applied at the genome-wide scale, performing well relative to other unsupervised methods [6].

While many of these approaches have relied on user-defined or truth set-based thresholds for determining the network, the correlation- and partial correlation-based methods can in principle calibrate established tests to a desired level of prediction accuracy via control of the false discovery rate (FDR) alone. However, such tests nominally assume independent and identically distributed (i.i.d.) microarray experiments. Work in differential gene expression analysis has demonstrated the necessity in such testing procedures of accounting for the correlation and consequent dependency inherent in microarray data, e.g., [7-9]. Potential sources of dependence between microarray experiments include biases in microarray preparation and lab-specific environmental factors (studied in the context of microarray reproducibility in e.g., [10,11]), and are particularly pertinent in compendia comprised of data from multiple labs. Moreover, recent work shows that the very gene-gene correlations in question in TRNI can even induce an *effective *dependency among seemingly homogeneous and independent sets of experiments (e.g., from a single project/lab). Thus, we expect such *effective *dependency (if not also real dependency) between microarray experiments, given that our approach to TRNI is based on the expectation of meaningful gene-gene correlations across the dataset. Such (effective) dependency invalidates the assumption of i.i.d. experiments upon which the statistical tests are based, thereby complicating the calibration of these tests.

To the best of our knowledge, the phenomenon of dependency in microarray compendia and its implications for TRNI has been noted and addressed only indirectly in the literature to date. In particular, it is known that the actual null hypothesis model in TRNI methods based on statistical testing typically will not conform to the nominal null model (i.e., that model suggested by standard theory), and that dependency is a possible culprit [5,12]. Furthermore, methods have been proposed to adaptively infer the form of that model from the data using, for example, principles of empirical null modeling (e.g., [12,13]). However, such methods do not facilitate quantification and exploration of the nature of this dependency in and of itself.

Here in this paper, we sought to explicitly quantify and characterize dependency, in the context of an *Escherichia coli *microarray compendium, containing both mRNA expression data and substantial information on experimental conditions. Then, utilizing the large set of known TF-gene interactions in *E. coli *from RegulonDB [14] to evaluate performance, we explored the implications of such dependency in TRNI using the correlation relevance networks method [1]. In doing so, we propose a new method of TRNI, which is simple but effective. On a broader scale, our contributions are aimed at lending a more quantitative structure to the discussion of optimal construction of mRNA expression compendia for TRNI.

Throughout this paper, as above, we refer to both "effective dependency" and "dependency". In seminal work [7], Efron has shown both empirically and theoretically that it is possible for microarrays to be statistically independent and yet, due to the very gene-gene correlations that are of interest in TRNI, these same arrays are *effectively *dependent, in the sense that empirical correlations among experiments can be inflated. Efron's focus in [7] was on the implications of this effective dependency on statistical tests for independence of a set of microarray experiments from a single project/lab in the context of differential expression analysis. He introduces an expression for the effective number of genes (which we denote *p*_*eff *_) that plays a key role in that work. In contrast, we focus here on the implications of dependency (effective or otherwise) on the task of TRNI, based on a compendium of microarray experiments, and introduce the natural complementary notion of the effective number of experiments (which we denote *n*_*eff *_).

Realistically, both types of dependency (i.e., effective and real) need to be dealt with in analyzing microarray compendia. The effective dependency is a given considering our expectation of gene-gene correlations, whether the experiments in the compendia are dependent or not [7]. But in fact, in addition, it is natural to expect that there also be actual dependence among experiments, whether due, say, to biases introduced by sample collection and/or microarray preparation, or to environmental variables that can vary between different laboratories or even projects within laboratories. Separation of effects of true dependency from those of effective dependency is complicated (e.g., see [7]) and is not our goal here. Rather, we aim only to accurately quantify the aggregate effects and, where necessary, adjust for them appropriately in TRNI analysis.

For the work detailed below, we utilized the Affymetrix *E. coli *compendium available on M3D, with mRNA expression data and corresponding experimental condition metadata for *n *= 376 experiments and *p *= 4298 "genes" (probe sets corresponding to coding sequences) (E_coli_v4_Build_5, [15]). This compendium is comprised of many sets of related experiments. Here, we define a "project" as a set of microarray experiments conducted under the same principle investigator towards investigation of related questions. In the majority of cases, a publication defined a project, but in some instances microarrays from multiple publications were combined to form one project.

Applying a test proposed in [7], the null hypothesis of i.i.d. experiments in the *E. coli *compendium was rejected, with visually evident structure based on project membership. Upon regrouping the data, we observed that experimental condition factors were a significant driving force behind the observed structure. We found that the vast majority of significantly correlated pairs of experiments were between experiments in the same project.

We explored the implications of this dependency between experiments in TRNI. This exploration was enabled by a summarization of the dependency in the form of an estimate of the effective number of experiments, *n*_*eff*_. A greedy search for microarray experiments that maximized *n*_*eff *_revealed that a larger *n*_*eff *_was achieved with a subset of the compendium, with the peak *n*_*eff *_attained using less than one third of the experiments. This surprising result suggested that subsets of the data might effectively contain more information than the full compendium. We used correlation as our measure of gene-gene interaction for TRNI (after comparing to other standard methods like partial correlation and CLR), and were able to evaluate performance in TRNI using the large set of known regulatory interactions in *E. coli *from RegulonDB [14]. Consistent with the observed peak in *n*_*eff *_, we found that subsets of the compendium also performed better than the full compendium in TRNI. Again, detailed examination of the data suggested that experimental conditions were a driving force behind the observed changes in *n*_*eff *_and TRNI performance.

We then used *n*_*eff *_to adjust p-values in tests for statistically significant edges and demonstrated that FDR levels using these values were within range of empirical FDR levels (derived from RegulonDB), while this was not the case when the actual number of experiments was used. Accurate computation of FDR levels for TRNI enables reliable predictions even when a truth set like RegulonDB is not available. We found that our TRNI method produced networks similar to those derived from RegulonDB-based thresholds. Using recent experimental findings, we confirmed the inferred topology of known TF Lrp, and examined that of a predicted hub in our network, putative TF YrbA.

## Results

### Structure and dependence in a compendium of microarrays

**Figure 1:**
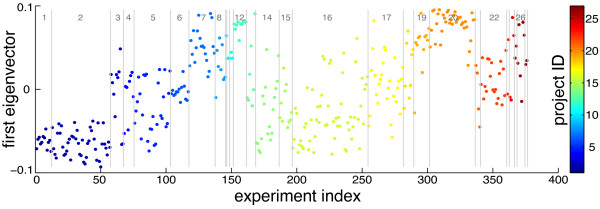
**Visualization of structure within the compendium**. The values of the first eigenvector, *v*1, of the experiment correlation coefficient matrix of the Affymetrix *E. coli *compendium plotted against their corresponding experiment index. Point color and gray numbers indicate project index, and gray lines also delineate projects.

We first performed permutation tests following methods in [7] to determine whether experiments in the *E. coli *Affymetrix microarray compendium were i.i.d. If the microarray experiments were indeed i.i.d., we would expect the components of the first eigenvector of the experiment correlation matrix, *v*1, to be random with respect to the experiment order. We plotted *v*1 against the experiment index with experiments grouped by project (Figure 1). Structure across these values was visually evident: in many cases, they were grouped by project. Results from permutation tests using a block statistic (see Additional file 1), with blocks defined on projects, strongly suggested the existence of structure within the data set, with p-value = 0 for 5000 permutations.

**Figure 2:**
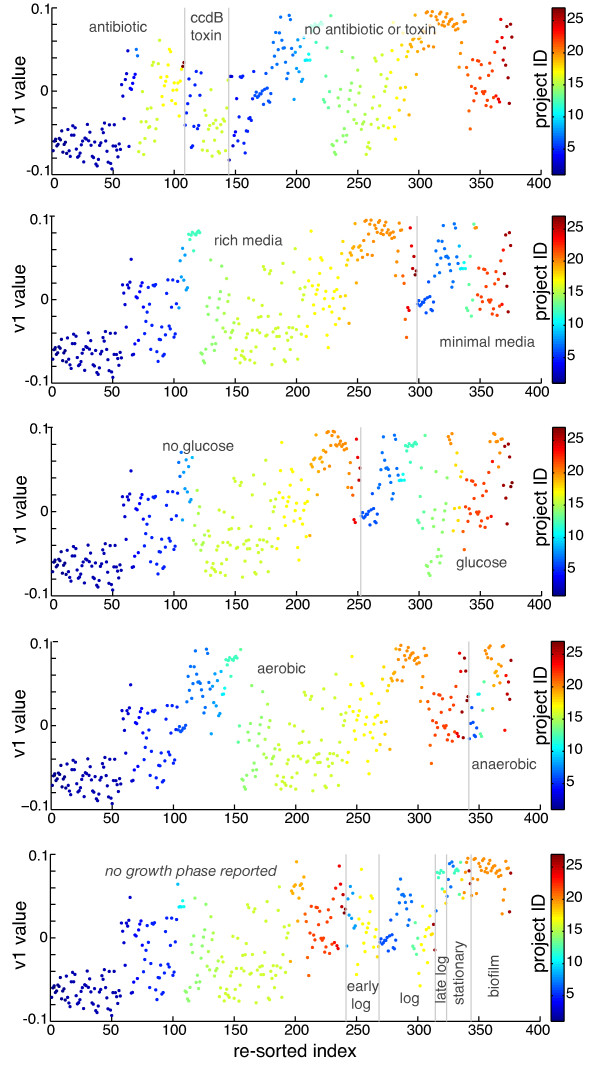
**Visualization of experimental condition-driven structure within the compendium**. The values of the first eigenvector, *v*1, of the experiment correlation coefficient matrix of the Affymetrix *E. coli *re-sorted according to experimental condition factors. Gray lines delineate groups, and groups are labeled by their condition factor in gray text on the plots. Point color indicates project index (same project coloring as in Figure 1).

In order to look at factors beyond project membership that may be contributing to the observed structure, we utilized the experimental condition data in M3D. We define an experimental condition factor as any detail about each microarray experiment that is part of the curated metadata on M3D; this includes any experiment variable that was reported for a microarray experiment in its associated publication, e.g., growth phase, strain, temperature, pH, culture type, etc. Assigning each experiment the *v*1 value corresponding to its index in the original order (as in Figure 1), we re-sorted the experiments according to a given experimental condition factor. For example, experiments in which glucose was present in the media were grouped together, and all those without glucose formed a separate group. Results for five experimental condition factors are shown in Figure 2. Indeed, we observed structure in each of these five cases across experiments from different projects, and two-sample *t*-tests in each case were rejected with p-values *<*1e-07. Note that in these tests, the antibiotic and ccdB toxin experiments (Figure 2, top panel) were grouped together, as ccdB is known to have a similar mechanism of action. Similarly, experiments with cells in late-log, stationary, or biofilm growth phase (Figure 2, bottom panel) were grouped and compared to all other experiments (whether growth phase was specified or not). Multi-way ANOVA analysis (Additional file 1, Supplementary Table S1) of the effect of these five experimental condition factors on their corresponding *v*1 values revealed that three of the condition factors (presence vs. absence of antibiotic/ccdB toxin, rich vs. minimal media, and early stage (and unspecified) vs. late stage growth phase) were likely influencing these values, while the structure observed for the other two condition factors (no glucose vs. glucose, aerobic vs. anaerobic) could likely be explained by the other factors. These results strongly indicate that the observed structure is *biologically *driven by experimental condition factors. From this perspective, with observation of structure due to individual condition factors, it is not surprising that we observe structure consistently at the project level (Figure 1), as the effects of these condition factors are undoubtedly confounded with project membership.

#### Correlated experiments

**Figure 3:**
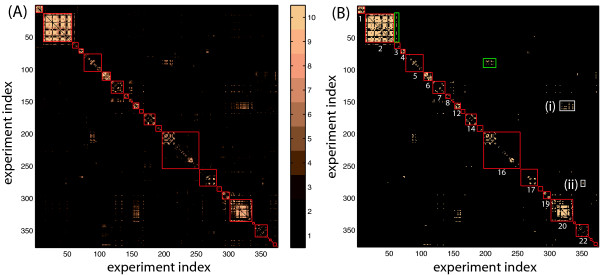
**Correlated experiments in the compendium**. Heatmaps of (A) -log(p-value) for experiment correlation coefficients (colorbar) and (B) significantly correlated experiments at FDR ≤ 0.1 (shown in gold). Red boxes delineate projects, with project index labeled below the boxes (where space permitted). Green and white boxes are referenced within the text.

Given the structure observed in the data set above, it was reasonable to ask which experiments in the compendium were significantly correlated. We assessed this following [7] using experiment-experiment correlation. This is not straightforward in the presence of dependency, and accordingly, the author in [7] used an estimate of the effective number of genes *p*_*eff *_(Equation 2) in testing for significantly correlated experiments. We found that *p*_*eff *_= 14.66 for this compendium, a drastic reduction compared to the number of genes *p *= 4298. Following [7], we used *p*_*eff *_as the sample size to calculate p-values in testing for correlation between experiments using Fisher transformed correlation coefficients (Figure 3A). Using the Benjamini and Hochberg FDR procedure (BH-FDR) [16] (Equation 10), we applied an FDR threshold q = 0.1 to these p-values and identified 1251 pairs of significantly correlated experiments (Figure 3B), constituting 2% of all possible pairs. Consistent with the trend observed in Figure 1, it is clear that a majority of these were pairs of experiments from the same project (in red boxes). We note that using the number of genes *p *= 4298 as the sample size in these tests resulted in 91% of all pairs significantly correlated at q = 0.1 (data not shown), a rather dubious value.

It is not surprising that the majority of correlated experiment pairs were from the same project. Many factors could contribute to such correlation, including similarity of experiments within the same project (i.e., a large set of shared experimental conditions), biases introduced by sample collection and/or microarray preparation, and environmental variables that can vary between different laboratories (and thus projects). We quantified the correlated-ness of projects as the fraction of correlated pairs (FCP) in each project. Considering only projects with more than three experiments, we observed that five of the six projects with the largest FCP (projects 1, 2, 6, 12, and 20) were predominantly comprised of genetic perturbation experiments, demonstrating that this type of perturbation yields generally less diverse expression profiles.

There were also several cases in which experiments from different projects were correlated. A full list and description of significant between-project correlations is included in Additional file 1, Supplementary Table S4. In some instances, these correlations would be expected due to similarity of experimental conditions, as was the case for the pairs in the green boxes (Figure 3B). However, pairs in the white boxes were not necessarily expected. In box (i), we see several correlations between experiments in projects 12 and 20, indicating that the high cell density (O.D. 595 nm *>*11) late log conditions in project 12 have similar expression profiles to several biofilm conditions in project 20. In box (ii), treatment with the antibiotic spectinomycin in project 17 correlated with serine hydroxamate treatment in a relA knockout in project 22. This indicates that serine hydroxamate treatment (used to induce stringent response) in cells unable to undergo canonical stringent response (due to deletion of relA) induces a similar transcriptional response to treatment with spectinomycin, an antibiotic known to act on the ribosome and inhibit protein synthesis. Finally, we observed that many experiments in which cells were sampled in the late-log, stationary, and biofilm growth phases tended to correlate with one another irrespective of other factors, including whether cells were grown in rich or minimal media, suggesting that these low-to-no growth state cells share similar expression patterns that don't vary significantly with perturbation. This is consistent with the clear separation of values corresponding to these later stages of growth (late-log through biofilm) observed in Figure 2, bottom panel.

Figures 1, 2, and 3 provide stark visual evidence of the dependency among experiments within the compendium. This type of analysis can be used to guide experiment design by identifying conditions that correlate across different projects (potentially indicating that surveying only a subset of the correlated conditions is necessary), and also highlighting projects with minimally correlated experiments (as examples of perturbation types and combinations that yield diverse transcriptional responses). Furthermore, unexpected correlations between well-studied and less well-studied experimental conditions can be used to gain insight into mechanisms of the resulting cellular response.

### Effective sample size and choice of experiments

We expected that the dependency between experiments would lead to an effective reduction in the number of experiments, just as [7] found that dependency between genes led to an effective reduction in the number of genes. Here, we were more naturally interested in the effective number of experiments, both as a potential measure of relative "usefulness" of experiments in the compendium, and also as it is relevant in testing for significant correlation between genes (e.g. in TRNI). Accordingly, we defined an expression for the effective number of experiments, *n*_*eff *_(Equation 4), analogous to that of *p*_*eff *_in [7]. We found that *n*_*eff *_was equal to *p*_*eff *_, as can be predicted by Theorem 1 in [7] (see Methods text and Equation 6), indicating that this quantity is essentially representative of a certain notion of reduced dimensionality for the entire data matrix.

**Figure 4:**
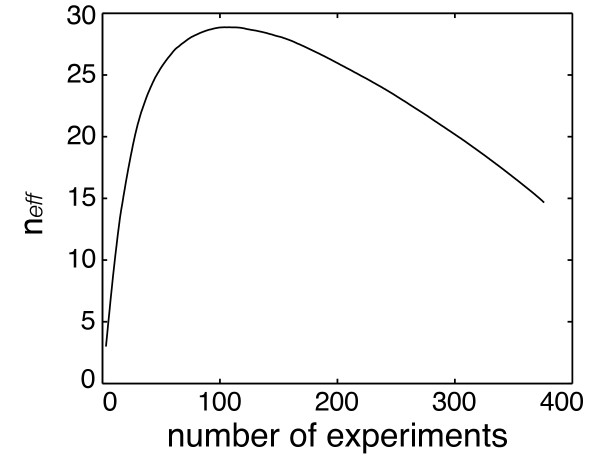
**Greedy search for experiments to maximize *n*_*eff*_**. Plot of effective number of experiments *n*_*eff *_vs. number of experiments. Experiments were added to the data set via a greedy search for experiments yielding maximum *n*_*eff *_.

Given the varying degrees of correlation between experiments observed above, we expected some experiments to be more informative than others. While it is challenging to address the broad issue of choice of experiments in its full generality, we propose here to use *n*_*eff *_as a metric of relative usefulness of experiments. We conducted a greedy search for combinations of experiments yielding maximum *n*_*eff *_. Experiments in the compendium were added one at a time until all experiments were included, at each step adding that experiment which maximized *n*_*eff *_. Interestingly, we saw a peak of *n*_*eff *_= 28.87 using 104 experiments, and a decrease in *n*_*eff *_as the remaining experiments were added (*n*_*eff *_= 14.66 using all 376 experiments) (Figure 4). As might be expected given the project-based structure observed in Figures 1 and 3, the subset of 104 experiments at the peak of this curve included experiments from nearly all projects in the compendium (25 of 27 projects).

The peak in the *n*_*eff *_curve indicates that adding experiments can decrease *n*_*eff *_. While at first glance this result may seem counter-intuitive, it is simple to construct a small-scale example to illustrate this phenomenon. Consider the case where our samples take the form of three scalar values *x*_1_, *x*_2_, and *x*_3_. Let *corr*_*ij *_be the correlation between samples *i *and *j*. If *corr*_12 _= 0.25, and *corr*_13 _= *corr*_23 _= 0.75, we find that *n*_*eff *_= 1.88 when only samples 1 and 2 are included, but *n*_*eff *_= 1.67 when all three samples are included. Thus, adding a third experiment with a strong enough correlation to both of the first two experiments effectively reduces the sample size. This is a toy illustration of the well-recognized role that latent variables can have in correlation-based analyses, wherein a subset of more fundamental variables can actually drive what appears to be more complex behavior among an ostensibly larger number of variables (e.g., [17]); sample 3 in this example clearly "drives" much of what is in samples 1 and 2. The same phenomenon is occurring in the microarray compendium, but at a much larger (and therefore more opaque) scale.

**Figure 5:**
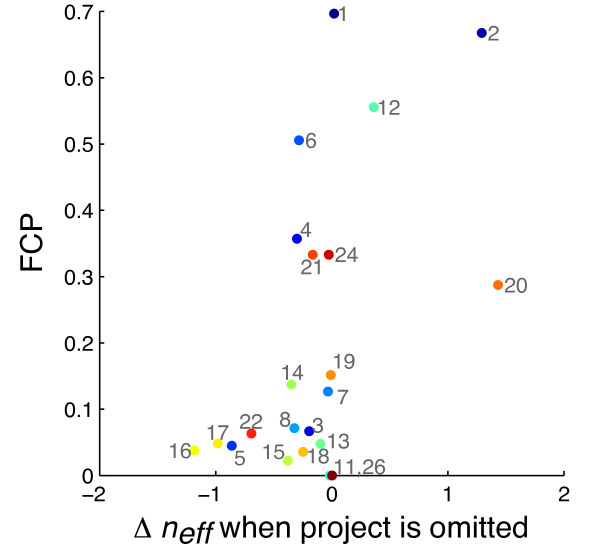
**Within-project correlation plotted against per-project contributions to *n*_*eff*_**. Fraction of correlated pairs (FCP) within a project vs. change in *n*_*eff *_when that project is omitted. Only projects with three or more experiments were included in the analysis.

We also evaluated *n*_*eff *_for subsets in which projects were omitted from the data set, one at a time; i.e for each project, we removed only that project from the full compendium, computed *n*_*eff *_, and looked at the change in *n*_*eff *_of the subset relative to *n*_*eff *_when all data were included. Similarly, we quantified the correlated-ness of each project as the fraction of correlated pairs (FCP) in that project (for FDR ≤ 0.1, as in Figure 3B). In Figure 5, we looked at FCP vs. change in *n*_*eff *_for all projects and found that projects with highly correlated experiments (large FCP) generally contributed less to or even decreased overall effective sample size. Omission of high FCP projects 2, 12, and 20 actually increased effective sample size; notably, all three of these projects were predominantly comprised of genetic perturbation experiments, indicating that this type of perturbation is prone to contributing redundant information to the data set.

### Implications for transcriptional regulatory network inference (TRNI)

**Figure 6:**
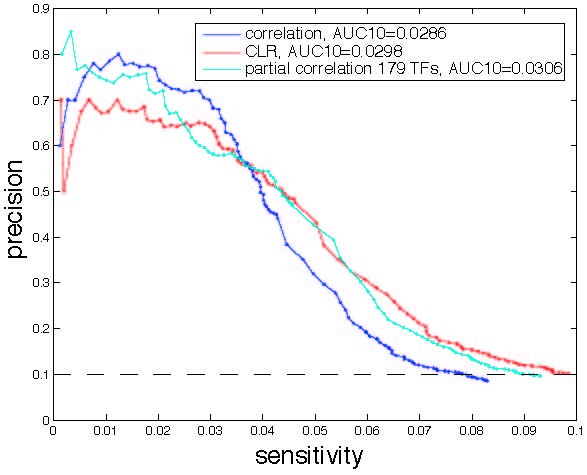
**Comparison of the performance of three TRNI algorithms**. Plots of precision vs. sensitivity for three TRNI methods applied to the *E. coli *compendium: correlation, CLR, and partial correlation. The partial correlation result used the set of 179 known TFs in RegulonDB as regressors. This was the best result observed for partial correlation in Additional file 1, Supplementary Figure S1A. AUC10 is the area under the precision vs. sensitivity curve but above 10% precision. The dashed black line indicates 10% precision.

We applied the concepts presented above 1) to evaluate contributions of subsets of experiments to TRNI accuracy and 2) for edge selection in TRNI. We used the set of known regulatory interactions in RegulonDB [14] to evaluate performance in TRNI. We focused on TRNI using correlation as our measure of interaction between two genes for two principal reasons. First, statistical testing of correlations is straightforward and well-established, in the standard case of i.i.d. measurements, with formulas for calibration of tests involving the sample size *n *in a straightforward fashion. This latter aspect in turn allows us to propose a rather simple and straightforward adjustment of the tests as applied for edge selection in TRNI, substituting the estimated *n*_*eff *_for *n *in the standard formula to account for dependencies in the data set. While analogous standard statistical tests exist for partial correlation, this method requires specification of the set of regressors; this can be nontrivial, with performance in TRNI substantially affected by the choice. (See Additional file 1 for an extended discussion of partial correlation results.) Second, we found that correlation performed similarly to partial correlation algorithms [4,5] and the mutual information-based context likelihood of relatedness (CLR) algorithm [6] for this Affymetrix *E. coli *compendium (Figure 6 and Additional file 1, Supplementary Figure S1). We observed that correlation performed as well or better than the other methods at higher precision (e.g. * >*55% precision), which is arguably of most interest given the goal of identifying highly probable edges. We emphasize that we do not adopt a correlation-based method because it is better, but rather because it i) performs similarly to arguably more sophisticated unsupervised pairwise inference methods (Figure 6), and ii) at the same time, is more amenable to our study of *n *and *n*_*eff *_.

#### Performance of subsets of the compendium in TRNI

**Figure 7:**
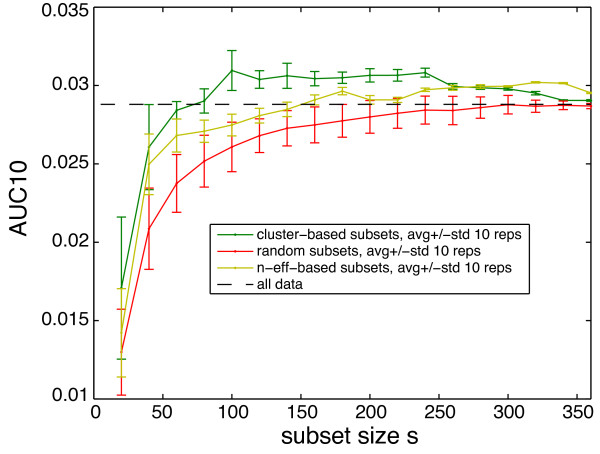
**Performance of subsets of the compendium in TRNI**. Correlation-based TRNI AUC10 with varying experiment subset size for three subset selection methods. The dashed black line marks the AUC10 using the full compendium.

Motivated by the observed peak in *n*_*eff *_using a subset of the compendium, we looked at the performance of subsets of the compendium in TRNI. Subsets were selected (i) randomly, (ii) based on the *n*_*eff *_greedy search, or (iii) via clustering as in [6] (one experiment from each cluster). Performance was measured as AUC10, the area under that part of the RegulonDB-based precision vs. sensitivity curve (e.g. curves in Figure 6) above 10% precision. (Note, the area below 10% precision was excluded to avoid computation of the long tail of these curves, which stretches out along the remaining range of sensitivity at very low precision, an arguably irrelevant precision range in our consideration of performance.) The results for subsets including 20 to 360 experiments are shown in Figure 7.

We found that subsets selected to maximize *n*_*eff *_uniformly outperformed randomly selected subsets and, for sufficiently many experiments (i.e., *>*150), outperformed the full compendium. We also observed that subsets selected to maximize *n*_*eff *_generally included experiments from more projects than random subsets at a given subset size (Additional file 1, Supplementary Figure S3). Performance using subsets selected via clustering was found to be the best, uniformly outperforming both random and *n*_*eff *_selected subsets for most sample sizes (i.e., *<*250), and outperforming the compendium with substantially fewer experiments (i.e., *<*80) than *n*_*eff *_selected subsets. This improvement is to be expected, as clustering is making more sophisticated use of the information in the data than *n*_*eff *_, with the former considering the distance (measured as correlation in this case) between all experiments simultaneously, and the latter considering only one experiment at a time and always searching for the experiment most distant from the subset already selected. Regardless, we believe these findings support the merit of *n*_*eff *_as a quantity relevant to the relative performance of subsets of experiments in TRNI. The improved performance using cluster-based subset selection is intriguing and may merit further study, but is beyond the scope of this work. However, we also note that when this comparison was conducted using alternative *E. coli *compendia, the outcome was more nuanced, with the *n*_*eff *_-based and cluster-based subset selection methods each outperforming the other over certain separate ranges of subset size (see [18], and Additional file 1, Section 8).

**Figure 8:**
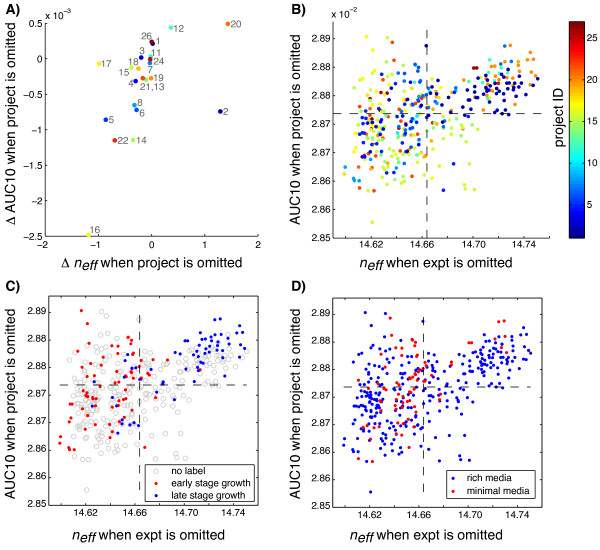
**Per-project and per-experiment contributions to *n*_*eff *_and TRNI performance**. (A) Change in AUC10 vs. change in *n*_*eff *_when project is omitted. Only projects with three or more experiments were included in the analysis. (B-D): Change in AUC10 vs. change in *n*_*eff *_when experiment is omitted, colored by (B) project ID, (C) growth phase stage state, or (D) media type state.

We also evaluated per-project change in *n*_*eff *_(as in Figure 5) and TRNI performance; i.e for each project, we removed only that project from the full compendium, computed *n*_*eff *_and AUC10 in TRNI, and looked at the changes of these measures relative to their values when all data were included. We found that changes in *n*_*eff *_roughly trended with changes in AUC10 (Figure 8A), indicating that projects positively contributing to overall *n*_*eff *_were also some of the most informative projects in overall performance in TRNI. Notably, project 16 contributed the most positively to TRNI accuracy; this is the largest project in the compendium, and includes time-series experiments with antibiotic and toxin treatments, all conducted in rich media. We found that two of the three projects that decreased overall *n*_*eff *_also decreased TRNI accuracy (projects 12 and 20), while project 2 contributed positively to TRNI performance. This could be attributed to the fact that project 2 is relatively large (second largest in compendium, 45 experiments), or possibly because the specific perturbations in this project (over-expressions in presence of antibiotic) are pertinent to regions of the transcriptional regulatory network that are 1) well-sampled in this compendium (so that they boost support for the edges that are inferable given the data) and/or 2) better represented in RegulonDB.

We repeated the same analysis on a per-experiment basis, where experiments were omitted one at a time from the data set and *n*_*eff *_and AUC10 were computed. We observed a moderate trend between per-experiment change in AUC10 with change in *n*_*eff *_(Figure 8B). This trend was stronger among experiments with positive change in *n*_*eff*_, i.e. less informative experiments. The weaker trend with AUC10 for experiments that were informative to *n*_*eff *_could reflect the fact that AUC10 is a measure of performance in a specific task (TRNI), and it is possible that certain experiments are informative generally but not necessarily informative in the specific task of TRNI (or, at least not informative in the inference of edges in RegulonDB, which we are using to assess performance). We also observed that all but one of the 45 experiments in project 2 fell in the upper right quadrant of this plot, in contrast to the overall positive per-project contribution to AUC10 observed for this project in Figure 8A, demonstrating that, while all of these experiments might be similar to one another (redundant to some degree), removing the entire project from the data set is detrimental.

In order to investigate whether specific experimental condition factors were more or less informative in these measures, we replotted Figure 8B coloring points based on their state in each of the five condition factor attributes considered in Figure 2 (e.g. Figures 8C and 8D). We then tested whether there was a relationship between the distribution of points and each binary condition factor (e.g. minimal media or rich media) by binning points into four bins based on overall AUC10 and *n*_*eff *_values (dashed black lines in Figures 8B, C, and 8D) and performing a chi-square test for independence (with the null hypothesis that values are distributed independent of the condition factor labels). Plots for the two condition factors with the smallest p-values in this test are presented here: growth phase stage (p-value *<*7e-12) and media type (p-value *<*3e-04) (Figures 8C and 8D, respectively). It is evident that experiments with early stage growth phase labels (early log and log) were generally more informative than those in the later stage growth phases (late log, stationary, and biofilm) (Figure 8C). Additionally, in Figure 8D we see that experiments conducted in minimal media were generally informative, though rich media experiments were not necessarily less informative (as we observed in the case of project 16, the most informative project in Figure 8A, which was conducted in rich media).

#### Edge selection for correlation-based TRNI using FDR

In *E.coli*, it is possible to evaluate TRNI performance and guide desired edge selection thresholds using RegulonDB, as we have done in our analysis above. However, in general, this is not the case; for most other species, it is necessary to select edges purely based on the data, without aid from a truth set. Edge selection in this context is increasingly important as transcription profiling (via RNA-seq) experiments from species with no known regulatory interactions accumulate in initiatives such as the Human Microbiome Project [19] and the Ten Thousand Microbial Genomes Project http://sz.genomics.org.cn/en/. Controlling the FDR in multiple hypothesis testing can be used to guide this process for TRNI [5]. FDR estimates rely on correct computation of p-values, which, in the case of the Fisher transformed correlation coefficients we use, depend on sample size *n *(see Methods). Thus, the effective reduction in sample size from *n *to *n*_*eff *_has critical implications in testing for correlations between genes in TRNI.

**Figure 9:**
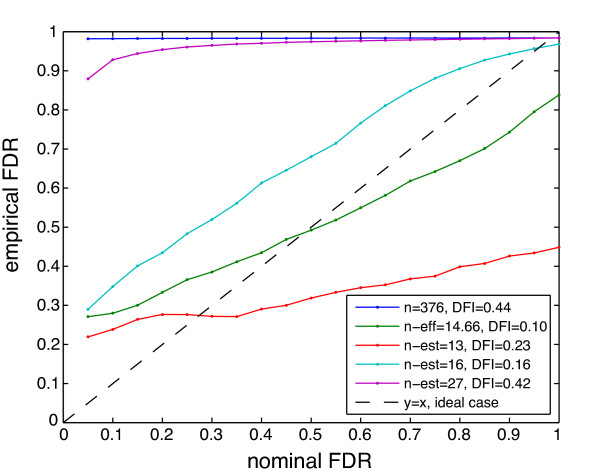
**Evaluation of BH-FDR control for correlation-based TRNI**. Empirical vs. nominal FDR for correlation-based TRNI. In all cases, correlation-based TRNI was applied to the full data set, with edge selection using nominal *n*, *n*_*eff *_, or other values for *n *(*n*_*est*_) to compute p-values for the BH-FDR analysis. The ideal case of y = x was included for comparison (dotted black line). Difference from ideal (DFI) was quantified as the area under a plot of the absolute difference between a given curve and the ideal case.

We computed gene-gene correlation *z*-values using the full data set and calculated corresponding p-values using two choices of sample size: i) the nominal number of experiments *n *= 376, and ii) the effective number of experiments *n*_*eff *_= 14.66, the latter of which adjusts for dependency among experiments (Equation 8). (Note that this is in contrast to, for example, Figure 4, where we were using *n*_*eff *_to select subsets of experiments.) For each set of p-values, we determined thresholds for desired FDR levels using BH-FDR ("nominal FDR"), and at these same thresholds, computed an "empirical FDR" using RegulonDB. (Note, this "empirical FDR" is more accurately described as the false discovery proportion (FDP), but we've chosen to use "FDR" to simplify our discussion.) Ideally, these two FDR values would be equal at each threshold, indicating that the nominal FDR levels accurately reflected the empirical FDR from the known truth set. (Note that this ideal case also depends on RegulonDB being a good representation of the truth; we address this point in the Discussion section.) The results are shown in Figure 9. There it can be seen clearly that using the nominal number of experiments *n *to compute p-values led to drastically inaccurate thresholds, according to RegulonDB, while using *n*_*eff *_yielded results within acceptable range of the ideal case (i.e., roughly along the 45 degree line). Results in Figure 9 were corroborated by histograms of the p-values for each case: p-values calculated using *n *were far from the expected uniform distribution, with a substantial majority of p-values near zero, while those using *n*_*eff *_were much closer to uniform (Additional file 1, Supplementary Figure S4).

Furthermore, and somewhat surprisingly, we observed that using values for *n *even slightly higher (*n *= 16) or lower (*n *= 13) than *n*_*eff *_yielded noticeably worse results (Figure 9), providing further evidence that these effective sample size estimates are meaningful quantities. Note, in particular, that a naive choice of sample size, such as the number of projects (i.e., 27), while vastly less than the nominal sample size, would still do little better than the nominal sample size. Overall, these findings support the use of BH-FDR for network edge selection when the effective sample size of the data set is taken into account. Additionally, the successful application of *n*_*eff *_in this setting substantiates the use of the equivalent *p*_*eff *_for identification of correlated experiments in Figure 3, where there was no truth set available for comparison.

Thus, in the combination of the correlation relevance networks approach and edge selection via FDR (taking *n*_*eff *_into account), we propose a simple and general method for TRNI (with steps enumerated below in the Methods section). The association measure of our method (i.e. correlation) performed comparably to other methods tested herein, and our method has the distinct advantage of providing meaningful estimates of the precision of predictions.

The most significant impact of using the corrected p-values for FDR-based edge selection in TRNI is a drastic reduction in the size of the inferred network. For example, using BH-FDR without our correction and setting FDR ≤ 20% (80% precision) leads to an overwhelming 244,460 interactions for which the null hypothesis is rejected (network edges), while, in stark contrast, using our correction at this same precision yields a network of 243 edges. Using RegulonDB-based empirical estimates of the true FDR, these networks correspond to 98% and 33% FDR, respectively, demonstrating that our correction yields a vast improvement. In Additional file 1, Supplementary Table S2, we summarized comparisons between networks defined by controlling FDR using either *n *or *n*_*eff *_or empirically estimating FDR using RegulonDB, with nominal or empirical FDR ≤ 20%, 40%, or 60%. Additionally, we looked at the inferred connectivity for the well studied TF Lrp to illustrate the validity of our approach at the gene level, using additional experimental data from [6] to evaluate inferred edges (Additional file 1, Supplementary Figure S5). Finally, we highlight a putative TF, YrbA, that is predicted to be a large hub by our approach, and present evidence that it is involved in regulation of translation (Additional file 1 text and Supplementary Figure S6).

On a final note, we point out that FDR analysis above utilized BH-FDR to determine p-value thresholds. However, BH-FDR is just one of many approaches for controlling/estimating FDR (summarized in [12]). Using the FDR evaluation framework afforded by RegulonDB for *E. coli *TRNI, we compared the performance of several FDR estimation methods available in the R package fdrtool[12] (Additional file 1, Supplementary Figure S7). We found that the performance offered by our simple approach, based on *n*_*eff*_, is comparable to the best performance observed by other tools, and noticeably better than some. See the Additional file 1 for details.

### Application of analysis to an additional data set

To gain further insight into concepts explored throughout this work, we compare and contrast our findings above to those obtained by applying the same analyses to an *E. coli *data set from Zare *et al. *[20]. These data are complementary in certain useful ways to those in the M3D compendium, in that they are small (only 46 conditions, rather than 376), from a single lab (rather than from multiple labs), and range across an intentionally diverse set of experimental conditions (rather than the often small variations across conditions of interest explored within labs contributing to the M3D data). This choice of data, and our findings, allow us to provide additional insight into how *n*_*eff *_may relate to the underlying biology in a data set.

The results of our analysis are presented in Additional file 1, Section 8. We observed that nearly all experiments in the compendium contributed positively to *n*_*eff *_, and peak *n*_*eff *_was very close to overall *n*_*eff*_. This indicates that when a compendium is designed in a more controlled manner with a goal of surveying a broad and diverse range of conditions, there is accordingly less redundancy (correlation) across the data set, in contrast to the M3D compendium analyzed herein. Congruous with this finding, we did not observe that subsets of the data consistently outperformed the full data set in the TRNI task. Notably, however, BH-FDR control of *n*_*eff *_-adjusted p-values for edges in correlation-based TRNI was not well-matched to empirical FDR estimates; we believe this is largely attributable to an overall lesser degree of informativeness of this compendium for correlation-based TRNI (paralleling similar observations made by Zare *et al. *[20]), and we discuss this further in the Additional file 1. Finally, we conducted a comparison of our *n*_*eff*_-maximizing selection of experiments to a measure of median gene set activity per experiment (proposed and computed for a subset of this compendium in [21]), which we argue can be expected intuitively to bear a reasonably strong correlation to each other, and found that this is indeed the case.

## Discussion and Conclusions

Having more microarrays in TRNI is not necessarily better. How many you have, and which you have, matters. While these statements arguably have been part of the common wisdom in this area for some time, our work here attempts to formalize and quantify relevant aspects of the basic issue of which and how many microarrays to use in TRNI. We have demonstrated the presence of dependency among experiments within a compendium of *E. coli *microarrays, and found that this dependency can be well-characterized by a corresponding effective sample size, *n*_*eff *_. We found that subsets of the compendium actually yielded larger *n*_*eff *_than the full data set, and correspondingly, these subsets performed better in TRNI. Finally, we proposed a straightforward method for TRNI that uses *n*_*eff *_in an explicit fashion, which performed comparably to competing methods and produced meaningful estimates of the precision of predictions. We emphasize that the merit of *n*_*eff *_derives from the totality of its role in this work, rather than from any single application. The fact that it can be seen to be usefully interwoven through various applications and quantitative summaries and analyses speaks strongly to its relevance.

A major result of the dependency in our compendium is a consequent sizable reduction of *n *to *n*_*eff*_, which suggests significant redundancy across the data set. Similar redundancy was observed in [6], using cluster analysis methods. There are many possible causes of this redundancy, including: the structure of the compendium, comprised of individual projects that often vary only one or a few experimental variables while controlling all others; the robust nature of the underlying biological network, making it difficult to perturb the system; a modular trend in transcriptional responses, resulting in similar expression changes over a range of conditions; or limitations of the microarray technology. Additionally, this result may indicate that the *in vitro *conditions that almost exclusively comprise this compendium only perturb a fraction of the transcipritonal regulatory network, promoting investigation of vastly different conditions, including *in vivo *and multi-organism cultures, as suggested in [6]. Apart from promoting exploration of entirely new experimental space, the analysis herein provides insight into potential strategies for experimental design, highlighting conditions that vary minimally across the compendium (e.g. cultures in biofilm or stationary growth phase) and sets of conditions (projects) yielding diverse expression responses (Figure 3).

Nonetheless, we note that while results presented in Figures 2 and 3 are highly suggestive of a link between experiment-experiment correlations and experimental condition factors, additional work would need to be done to establish this more rigorously. For example, one could conduct a design-based study to test for and quantify the effects of experiment-experiment interactions. Our work here is meant to lay the motivation for and suggest the need for additional work of this nature.

The TRNI approach proposed here follows a framework with some similarities to the GGM method proposed in [5], which also employed FDR estimation for network selection (using fdrtool), but used partial correlation instead of correlation. In our analysis, we found that this GGM method did not perform as well in TRNI on our data, and that corresponding FDR estimates deviated significantly from RegulonDB-based FDR estimates (see Additional file 1, Supplementary Figure S1).

As a peripheral benefit, our work demonstrated the utility of the RegulonDB-based testing framework used in [6] for evaluation of different methods of FDR estimation designed for high-dimensional data. Nevertheless, while RegulonDB provides a useful framework for evaluation of TRNI performance and FDR estimation using experimental data, this is still not an ideal test setting. RegulonDB is incomplete; the test set used for the Affymetrix *E. coli *compendium included interactions involving 1838 genes, less than half of the 4000+ genes predicted in *E. coli*. This truth set also potentially includes incorrect interactions, as it is manually curated and derived from experiment-based conclusions. Thus, these drawbacks should be considered when drawing conclusions from such evaluations, cautioning distinctions between methods that perform similarly. Nonetheless, we believe that this testing framework is valuable, particularly given that generation of truly representative simulated data sets is challenging due to the multi-level nature of the underlying biological network. Additionally, the accuracy of RegulonDB-based precision estimates in TRNI was supported by experimental validation carried out in [6].

The TRNI approach highlighted here provides a simple but general method for predicting highly probable transcriptional regulatory interactions from large collections of microarray data. This generalized approach can be readily applied to less well-studied organisms for which large microarray compendia are available, such as *P. aeruginosa *(348 samples for platform GPL84 in Gene Expression Omnibus (GEO) database [22]), and *S. oneidensis *(207 experiments in M3D [15]). Additionally, as RNA-seq and other improved methodologies become more widespread and begin to replace DNA microarray experiments, observations from microarray compendia can serve as useful tools, including guidance in experimental design as noted above. Also, it is highly likely that issues of dependency within novel-platform data sets will persist given the nature of the underlying biological network, so that considerations of such issues here will be applicable in this new context.

The work in this paper, taken as a whole, is aimed at the broader goal of providing a more quantitative framework for the discussion of the construction of microarray compendia for TRNI. We see the ultimate goal in this context to be the development of a complete, unified methodology for the design and use of compendia for TRNI - from choice of the experiments run, to assembly of the compendia, to the actual network inference.

## Methods

### Microarray compendium

Analysis was conducted using the RMA-normalized [23] E_coli_v4_Build_5 compendium of Affymetrix *E. coli *ASv2 microarrays available on M3D ([15], http://m3d.bu.edu). This data set included *p *= 4298 genes and 725 microarrays surveying *n *= 376 experimental conditions ("experiments") from 27 projects of microarray experiments, with projects defined as in the Background (see Additional file 1, Supplementary Table S3for the list of projects). Only the closest-to-average (CTA) replicate from each experiment was included in analysis (see Additional file 1 for details and comparison to other treatments of replicates in Additional file 1, Supplementary Figure S2).

### Double standardization of the data

All results presented in this work use double standardized data, unless otherwise specified. Double standardization of the RMA-normalized gene-by-experiment data matrix was carried out following [7]. The data were iteratively centered and scaled resulting in rows and columns with mean 0 and variance 1. First, the data were demeaned by centering the columns and then the rows. Second, the columns and then the rows were scaled. Demeaning and scaling were repeated until the difference between each entry of the data matrix in consecutive iterations was less than 0.01. This occurred in six iterations for this compendium.

We note that this double standardization is important, as we found that correlation-based TRNI performed better using double standardized data (i.e. double standardization applied to RMA-normalized data) than non-standardized data (i.e. RMA-normalized data) (see Additional file 1, Supplementary Figure S2). In addition, the theory described below assumes this double standardization, which simplifies the mathematics considerably.

### Calculation of effective sample size: *p*_*eff *_and *n*_*eff*_

Denote the doubly standardized data matrix by *X*, where *X*_*ij *_is the value corresponding to the *i*-th gene in the *j*-th experiment. Let Σ and Δ be the true gene-gene and experiment-experiment correlation matrices, respectively. Similarly, let $\hat{\Sigma }$ and $\hat{\Delta }$ be the sample gene-gene and experiment-experiment correlation matrices, respectively.

Since the entries of *X *all have zero mean and unit variance, we can calculate the matrix of gene-gene sample correlations as $\hat{\Sigma }=XX^{\prime }/n$ and similarly, the matrix of experiment-experiment sample correlations as $\hat{\Delta }=X^{\prime }X/p$. The entries of these matrices are just averages and, as averages, we would expect under standard i.i.d. assumptions that their variances would behave like $\text{Var}({\hat{\Sigma }}_{ii^{\prime }})\propto n^{-1}$ and $\text{Var}({\hat{\Delta }}_{jj^{\prime }})\propto p^{-1}$. When dependency is present, however, this behavior will not hold.

The actual form of these variances will depend on the full joint distribution of the values in *X *(of which, note, Σ and Δ are only marginal correlation matrices). To simplify calculations, Efron [7] assumes a matrix normal distribution

(1) $$X∼N_{p,n}(0,\;\Sigma ⊗\Delta ),$$

imposing a tensor-product form on the overall covariance matrix, and shows that it then follows that $\text{Var}({\hat{\Delta }}_{jj^{\prime }})\propto p_{eff}^{-1}$, where

(2) $$p_{eff}=\frac{p}{1+(p-1)\alpha^{2}}$$

and α is the total correlation

(3) $$\alpha^{2}=\sum_{i<i^{\prime }}\left(\Sigma_{ii^{\prime }}^{2}/\Sigma_{ii}\Sigma_{i^{\prime }i^{\prime }}\right)/\left(\begin{array}{c} p\\ 2 \end{array}\right).$$

Following standard practice, the author interprets *p*_*eff *_as an effective sample size i.e., the effective number of genes. Note that if there is no correlation among genes, α would be zero, and *p*_*eff *_= *p*, as is true in the classical i.i.d. case. Otherwise, *p*_*eff *_*< p*, indicating effectively fewer genes than nominal. In our empirical work, we used the sample correlation matrix $\hat{\Sigma }$ as an estimate for Σ to calculate the empirical estimate ${\hat{p}}_{eff}$ of *p*_*eff *_reported in our analyses; we have dropped the 'hat' notation in the main text for expository purposes.

Employing the same argument as above, but switching the roles of genes and experiments, in this work we argue analogously that $\text{Var}({\hat{\Sigma }}_{ii^{\prime }})\propto n_{eff}^{-1}$, where

(4) $$n_{eff}=\frac{n}{1+(n-1)c^{2}}$$

with

(5) $$c^{2}=\sum_{j<j^{\prime }}(\Delta_{jj^{\prime }}^{2}/\Delta_{jj}\Delta_{j^{\prime }j^{\prime }})/\left(\begin{array}{c} n\\ 2 \end{array}\right).$$

The value *n*_*eff *_is to be interpreted as a measure of effective sample size i.e., in this case, the effective number of experiments. If there were no correlation among experiments, *c*^2 ^would be zero, and *n*_*eff *_= *n*. Otherwise, *n*_*eff *_*< n*. We used the sample experiment covariance matrix $\hat{\Delta }$ to estimate Δ in calculating the empirical estimates ${\hat{n}}_{eff}$ of *n*_*eff *_reported in our analyses; again, we have dropped the 'hat' notation in the main text for expository purposes.

We note that neither the definition of *n*_*eff *_nor *p*_*eff *_is motivated by biology. Rather, they are mathematically motivated and applied in order to quantify dependencies between rows and columns of the data set. The definition of effective sample size through the scaling of variances is standard in statistics, particularly in topic areas involving dependent data, such as time series analysis or spatial data analysis. The nature of the effective sample sizes given above derives in part from the model in Equation 1 and, in particular, the tensor form of its covariance. The latter is an assumption that simplifies the mathematics and yields a closed-form expression, which facilitates interpretation (an important theme we emphasize throughout). Of course, it is possible that for a particular dataset the model assumptions used here may be too simplified, in which case the accuracy of *n*_*eff *_and *p*_*eff *_as effective sample sizes presumably will be affected. However, analyses like the FDR calibration study (summarized in Figure 9) suggest that, for the data used here, these definitions of effective sample size are largely on target.

#### Equality of ${\hat{p}}_{eff}$ and ${\hat{n}}_{eff}$

Theorem 1 of Efron [7] states that the empirical average and variance of the gene-gene correlations in $\hat{\Sigma }$ will be equal to those of the experiment-experiment correlations in $\hat{\Delta }$. Because in calculating *p*_*eff *_and *n*_*eff *_on real data, we substitute the sample correlation matrices $\hat{\Sigma }$ and $\hat{\Delta }$ for the true correlation matrices Σ and Δ in Equations 3 and 5, the implication of Efron's theorem is that necessarily

(6) $${\hat{\alpha }}^{2}=\frac{p[1+{\hat{c}}^{2}(n-1)]-n}{n(p-1)},$$

where ${\hat{\alpha }}^{2}$ and ${\hat{c}}^{2}$ are the empirical versions of *α*^2 ^and *c*^2^, respectively. Plugging this value in for *α*^2 ^in the expression for *p*_*eff *_in (2), we find that necessarily ${\hat{p}}_{eff}={\hat{n}}_{eff}$.

### Correlation-based TRNI using *n*_*eff*_

Our algorithm for correlation-based TRNI using *n*_*eff *_is a variation on the correlation relevance networks approach proposed in [1], in which the matrix of gene-gene correlation coefficients is used as the measure of interaction between genes, with a threshold applied to define the inferred network.

1. Compute the double-standardized data matrix *X*

2. Compute the matrix $\hat{\Sigma }=XX^{\prime }/n$ of gene-gene correlation coefficients

3. Apply the Fisher transformation to $\hat{\Sigma }$ i.e.,

(7) $$z_{ii^{\prime }}=\frac{1}{2}log\frac{1+{\hat{\Sigma }}_{ii^{\prime }}}{1-{\hat{\Sigma }}_{ii^{\prime }}}.$$

4. Compute *n*_*eff *_-adjusted p-values, by comparing the *z*_*ii'*_to a N (0,(*n*_*eff*_-3)^-1^) distribution.

5. Determine the interaction network by thresholding the p-values, using BH-FDR to control FDR at a specified level, and then reporting only putative TF-gene interactions

In the standard i.i.d. setting, the correlations ${\hat{\Sigma }}_{ii^{\prime }}$, between genes *i *and *i' *, would nominally have asymptotically normal distributions, with means Σ_*ii' *_and variances ${\left(1-\Sigma_{ii^{\prime }}^{2}\right)}^{2}/n$. Fisher's transform is a standard normalizing and variance stabilizing transformation, yielding values *z*_*ii' *_that are asymptotically normal with means 0.5 log[(1 + Σ_*ii'*_)/(1 - Σ_*ii'*_)] and common variance (*n *-3)^-1^. Under the null hypothesis of no correlation between genes *i *and *i*'(i.e., Σ_*ii' *_= 0), the relevant null distribution becomes simply a N (0,(*n*-3)^-1^) distribution.

In our setting, dependency in the data changes the distribution theory. It is nontrivial to capture these changes in closed-form, motivating an approximation using lower order moments. The mean will remain zero, under the null hypothesis, and empirical examination of our data suggests that the normal distribution is not an unreasonable approximation to the shape of the null. Furthermore, in the spirit of empirical null modeling (e.g., [12,13]), we find that substituting *n*_*eff *_for *n *in the nominal variance formula to be quite effective. (This type of simple substitution was also used in the test for experiment-experiment correlations adopted from [7].) The resulting null distribution used is thus

(8) $$N(0,\;{\left(n_{eff}-3\right)}^{-1}).$$

Two-sided p-values were calculated using this distribution.

We used the approach in [16] (herein referred to as BH-FDR) for controlling FDR in the simultaneous testing across TF-gene pairs. In this approach, for *m *tests, all *m *p-values are placed in ascending order,

(9) $$p_{1}\leq p_{2}\leq \ldots \leq p_{m}$$

and for all tests with p-values for which

(10) $$p_{k}\leq q\frac{k}{m}$$

the null hypothesis was rejected, where *k *is the index of the ordered p-value, and *q *is the desired FDR level.

Note, to evaluate FDR estimates for correlation-based TRNI, we compared nominal FDR to empirical FDR. The nominal FDR (based on BH-FDR) was used to determine the threshold defining predicted edges, and empirical FDR (the fraction of predicted edges that were false, 1-precision) was subsequently computed using RegulonDB ([14]; RegulonDB is described below).

### Correlation-based TRNI, performance assessment, and subset analysis

We compared correlation-based TRNI to partial correlation methods (including the graphical Gaussian model (GGM) method proposed in [5]) and the context likelihood of relatedness (CLR) algorithm [6] as described in Additional file 1.

Performance in TRNI was assessed using the set of known genetic regulatory interactions in RegulonDB version 6.2 [14]. When mapped to the genes in the Affymetrix *E. coli *compendium, this version of RegulonDB consisted of 5161 interactions involving 176 TFs and a total of 1838 genes. For all performance assessment, we only considered the 1838 × 176 entries of the gene-gene interaction matrix (inferred in each method) corresponding to the genes and TFs in this version of RegulonDB (though note that the full gene-gene interaction matrix was used to select FDR-based thresholds). We used the precision vs. sensitivity (recall) curve rather than the receiver operating characteristic (ROC) curve, as our focus was on reliable prediction of (potentially new) edges rather than recovery of known interactions. Precision was computed as the fraction of predicted edges that were true (1-FDR), and sensitivity as the fraction of true edges that were correctly predicted. To summarize performance of the methods in precision vs. sensitivity, we computed AUC10, defined as the area under the precision vs. sensitivity curve but above 10% precision.

We also assessed TRNI performance for subsets of the compendium. These subsets were selected in three ways: randomly, based on *n*_*eff *_, or based on clustering. For *n*_*eff *_, multiple experiment orderings were determined by conducting the greedy search to maximize *n*_*eff *_starting from different random seed sets of 10 experiments (as opposed to the full greedy search conducted in Figure 4). An *n*_*eff *_subset of size *s *was the first *s *experiments from a given *n*_*eff *_greedy search. Cluster-based subsets of size *s *were selected as in [6], where experiments were clustered into *s *clusters using correlation as the distance measure, and one experiment was selected from each cluster.

## Authors' contributions

EK conceived of the study; EC, TG, and EK designed the study; EC conducted the data analysis; EC and EK interpreted the results, and drafted the manuscript. All authors read and approved the final manuscript.

## Supplementary Material

Additional file 1 **Supplementary Materials**. Supplementary methods, results, figures, and tables that augment the work presented here, as referenced throughout this text. Click here for file
