# Supplementary material for: On the Choice and Number of Microarrays for Transcriptional Regulatory Network Inference
Source: BMC Bioinformatics. 2010 Sep 9;11:454. doi: 10.1186/1471-2105-11-454 (PMC2949888; doi:10.1186/1471-2105-11-454)
Supplement: Additional file 1 — Supplementary Materials. Supplementary methods, results, figures, and tables that augment the work presented here, as referenced throughout this text. [file 1471-2105-11-454-S1.PDF]

Supplementary Materials

Cosgrove, Gardner, and Kolaczyk

August 30, 2010

1 Supplementary Methods

1.1 Treatment of replicate microarrays

When a given experiment had more than one replicate, we only included the closest-to-average (CTA) replicate in analysis. The CTA replicate was the microarray in a set of replicates that was closest to the average of that set of replicates (minimum sum of absolute differences between the replicate and the average of replicates). If there were only two replicates for an experiment, one was chosen at random. TRNI performance using CTA replicate data was compared to two other treatments of replicates (including all chips or averaging replicate chips) in Supplementary Figure S2. We chose to use the CTA replicates for analysis in this work, as the CTA data performed better in correlation-based TRNI than including all replicates, and this approach avoids possible smoothing effects due to averaging.

1.2 Permutation tests for i.i.d. experiments

We conducted permutation tests for i.i.d. experiments following [2]. The $n \times n$ experiment correlation matrix was computed for the double standardized compendium. The first eigenvector of this matrix, $v1$, was calculated via singular value decomposition. If the experiments were indeed i.i.d., the order of the values in $v1$ should be random with respect to the order of the experiments. To test this, a block statistic

$$S(v) = v'Bv \tag{1}$$

was computed for $v = v1$ and 5000 random permutations of $v1$, $v = v1^*$. The block operator B was the $n \times n$ matrix:

$$B = \sum_h \beta_h \beta_h' \text{for } h\text{=1:number of projects} \tag{2}$$

where β_h is the $n \times 1$ vector:

$$\beta_h = \begin{cases} 1 & \text{for } j \text{ in project } h,\ j\text{=1:}n \\ 0 & \text{otherwise.} \end{cases} \tag{3}$$

The p-value of the test was then computed as the fraction of $S(v1^*)$ exceeding $S(v1)$.

2 Correlated pairs of experiments from different projects: Supplementary Table S4

Key similarities and differences between experimental conditions for correlated pairs of experiments (Expt A and Expt B) from different projects are presented in Supplementary Table S2, as well as the experiment names and the associated project ID. Row colors highlight groups of interesting/ potentially unexpected cases of correlation: between projects 12 and 20 (cyan), involving antibiotic treatments (red), involving stationary and biofilm growth phases (yellow), and a miscellaneous group (gray). Discussion of select correlations is included in the Results within the main text.

3 Comparison of TRNI methods

We compared the performance of three commonly implemented unsupervised methods for TRNI: correlation (i.e. correlation relevance networks [1]), partial correlation, and mutual information-based context likelihood of relatedness (CLR) [4]. The correlation method simply used the matrix of gene-gene correlation coefficients as the measure of interaction between genes, with a threshold applied to define the inferred network. The same was true for the partial correlation approach using partial correlation coefficients. However, this method requires specification of the set of regressors; this can be nontrivial, with performance in TRNI substantially affected by the choice (discussed below). A complete summary of the results using partial correlation is presented in the following section below. CLR was implemented using number of bins nbins=10 (CLR parameter 'n'), spline degree k=3, and method 'normal'. We observed that all three approaches performed similarly in TRNI (main text Figure 6), and that correlation performed as well or better than the other methods for higher precision (e.g. >55% precision).

4 Evaluation of partial correlation-based TRNI and associated FDR estimation

For comparison, we implemented TRNI and associated FDR control/ estimation methods using partial correlation as the measure of association. This approach depends on the specified set of regressors; following [7], Ch. 7, we used the set of 179 known TFs (according to RegulonDB) as the set of regressors ("179 TFs"). (Note, we also considered regressor sets that included putative TFs in addition to known TFs, but TRNI performance using these sets was markedly worse (2- to 9-fold decrease in AUC10) and therefore this analysis is not presented here.)

We also implemented the graphical Gaussian model (GGM) method proposed in [9], which utilizes a shrinkage estimator of partial correlation, where the set of regressors included all p-2 genes not included in the gene-gene interaction in question ("GGM"). Herein, we implemented this method using the R package `GeneNet`, which has replaced the now obsolete R package `GeneTS` that implemented the original method in [9]. Among other changes from the original method, `GeneNet` uses the R package `fdrtool` [10] to assess significance of network edges. We observed that the GGM method did not perform as well as the 179 TF set in TRNI (Supplementary Figure S1A).

We compared RegulonDB-based (empirical) FDR to BH-FDR based on p-values computed using nominal n for partial correlations based on the 179 TF regressor set. (In the i.i.d. case, Fisher-transformed partial correlations have a normal distribution similar to correlation, except

that the number of regressors, k, affects the distribution: $\mathcal{N}(0, (n-k-3)^{-1})$.) We observed that the corresponding empirical vs. nominal FDR curve deviated from the ideal case (Supplementary Figure S1B), similar to when n was used in the analysis in Figure 9. We also applied a method from `fdrtool` for estimating FDR (input: partial correlation; cutoff.method="fndr"; FDR type="lfdr" (local FDR)), which yielded a marked improvement over results using n (Supplementary Figure S1B, green curve).

We also evaluated the FDR estimates output by the GGM method via `fdrtool`, which included both tail area-based ("qval") and local ("lfdr") FDR estimates. In both cases, the results were far from the ideal curve (Supplementary Figure S1B, dashed curves). This deviation indicates that the FDR methods employed are not well suited for the shrinkage estimate of partial correlation used in the GGM method. Supplementary Figure S1C is a histogram of the `fdrtool` p-values (upon which the GGM (`fdrtool`) FDR curves in Supplementary Figure S1B are based), depicting a very large peak of near-zero p-values that is likely driving the high empirical FDR across the full range of estimated FDR. This is consistent with the large number of "significantly non-zero coefficients" found in [9], which prompted the authors to caution the direct application of these FDR thresholds.

5 Effects of data pre-processing, compendium version, and truth set version on performance in TRNI

Overall performance observed in main text Figure 6 was noticeably worse than previously observed for correlation and CLR in [4], prompting us to look at the source of this change. We compared performance of these two algorithms using two versions of the *E. coli* microarray compendium available on M3D (Build 1 and Build 5, 189 and 376 experiments, respectively), with two versions of RegulonDB (version 4 and version 6.2, 3102 and 5161 interactions, respectively) (Supplementary Figure S2). We determined that the noted drop in performance was largely attributable to use of the newer version of RegulonDB, indicating that many of the recently added regulatory interactions in RegulonDB are not captured by these microarray compendia. We found that performance improved from older to newer versions of the compendium, though this improvement was modest relative to the near doubling of the number of experiments that occurred in going from one version to the other. Additionally, we tested correlation and CLR performance for different pre-processing of the data, including whether or not double standardization was applied to the data, and how replicate microarrays were included (Supplementary Figure S2). Interestingly, we found that correlation performed better using double standardized data (i.e. double standardization applied to RMA-normalized data) than non-standardized data (i.e. RMA-normalized data). In fact, when data were not double standardized there was no clear improvement in going from the older version of the compendium to the newer. The improvement using double standardization can likely be attributed to standardization across genes, which minimizes effects due to differences in the variance of individual genes [2], while RMA-normalization does not include this gene-wise standardization [6]. In contrast, there was little difference in CLR performance with or without double standardization of the data. We believe this is due to the fact that CLR effectively standardizes on genes as well by using gene-specific conditional empirical densities in assigning z-values. Finally, we observed that including the average of replicates or the CTA replicate (see Methods) for a given experiment yielded slightly improved performance compared to including all replicates individually.

6 Examination of networks and edges inferred using FDR for edge selection

As mentioned in the main text, the most significant impact of using our FDR p-value correction for edge selection in TRNI, as opposed to using nominal n, is a drastic reduction in the size of the inferred network. Supplementary Table S2 presents summarizing comparisons between networks defined by controlling FDR using either n or n_{eff} (left and center columns, respectively), or empirically estimating FDR using RegulonDB (right column). The table includes a summary for networks at nominal or empirical FDR $\leq$ 20%, 40%, or 60%. (See Legend following Table S2 for complete description of table.) It is immediately apparent that using n yields unreasonably large networks (and likewise unreasonably low correlation thresholds), often involving nearly all genes included in the analysis, while using n_{eff} yields networks much more similar to those that are defined based on RegulonDB (empirical) estimates of FDR.

If we redefine the same networks using transcription units (TUs) as the nodes, we see a reduction in the size of the network (Table S2, "TU only" columns). This reduction (often around 50%) is consistent with the reduction in the number of possible nodes (4298 genes map to 2625 TUs), and is also reflected in the number of known interactions in RegulonDB (5161 known interactions in RegulonDB reduces to 2225 interactions between TUs, including 46 self-regulating TUs). The TU-only results in Table S2 indicate that our predictions aren't merely identifying TF-gene pairs that are transcribed together.

The bottom rows in Table S2 track the number of edges for three specific transcription factors (TFs) across the various networks. TF node Lrp is depicted in Supplementary Figure S5 (edge colors described in caption). We see that, in general, this hub looks similar in the RegulonDB-based FDR networks and the networks defined following our method for FDR control (left columns), and that precision of edges for this node based on experimentally validated edges (from [5] and [4]) match reasonably well with the corresponding FDR control level. We note that target genes for this node are most significantly enriched for a relevant GO term with nominal FDR$\leq$40% (using our approach): cellular amino acid biosynthetic process, p-value$\leq$1e-05.

Table S2 also tracks the number of edges for the putative TF YrbA across the various networks. This TF was predicted to be a large hub in our inferred networks. Using the FDR$\leq$40% network based on our approach, we found that the predicted target genes of YrbA were significantly enriched for translation (p-value$\leq$1e-34, including 41 genes that map to this term). Notably, 27 of the 123 targets inferred for YrbA were also inferred targets of the global regulator Fis, known to regulate the expression of many genes involved in translation. While this could indicate that *yrbA* is simply a gene that is regulated by Fis, this notion is countered by the fact that 1) YrbA and Fis are not predicted to regulate each other, and 2) only a fraction of the predicted YrbA targets are shared by Fis, while Fis is only predicted to regulate four other genes.

In order to learn more about how projects and experimental conditions were contributing to some of the observed correlations for *yrbA* expression, we looked at the expression plots for *prfB* vs. *yrbA* (corr=0.91) and *mreB* vs. *yrbA* (corr=0.81) (Supplementary Figure S6). We see that higher level expression of these genes are consistent with earlier stage growth phase (early log or log), and also that most of the experiments involving exposure to toxin or antibiotic resulted in higher expression of these genes. This could make sense given the predicted involvement of YrbA in translation, as translation decreases in later stage growth phases relative to earlier stage growth. These findings implicate YrbA as an interesting target for further study.

7 Comparison to other FDR methods

The FDR analysis presented in the main text utilized BH-FDR to determine (adjusted) p-value thresholds. However, as mentioned in the main text, BH-FDR is just one of many approaches for controlling/ estimating FDR (summarized in [10]). Using the FDR evaluation framework afforded by RegulonDB for *E. coli* TRNI, we compared the performance of FDR estimates derived using the R package `fdrtool` [10]. While there are many FDR estimation methods available, we considered this particular tool as it brings a number of the best features of various methods together. This method has three semi-parameters: input statistic (e.g., correlation or z-values), cutoff method ("fndr" or "locfdr"), and FDR type (tail area-based ("qval") or local ("lfdr") FDR). We found that using cutoff.method="fdnr" yielded consistently improved results compared to using cutoff.method="locfdr" (data not shown), and thus results presented here use cutoff.method="fdnr". Using the parameter settings most comparable to our n_{eff}-adjusted BH-FDR method, i.e. input statistic="normal" (z-values) and FDR type="qval" (tail area-based FDR), we observed that this FDR estimate did not match the ideal case as closely as our method (Supplementary Figure S7).

We also evaluated other semi-parameter settings for `fdrtool`, and found that using correlation as the input statistic ("correlation") and looking at local FDR results ("lfdr") produced estimates very close to ideal (DFI=0.05). The greatest deviation from ideal for this result was at low FDR; given the curves in main text Figure 6 (where precision never exceeds 80%), it was clear that the empirical FDR would not be lower than 20%, so it is not surprising that nominal FDR values did not match up in this region.

8 Application of analysis to an additional data set

To gain further insight into concepts explored throughout this work, we applied our analyses to the data set from Zare *et al.* [11]. This data set consists of microarray data surveying 46 diverse experimental conditions in *E. coli*, all conducted by the same lab using a cDNA microarray platform (p=4065 genes) (experimental conditions listed in Supplementary Table S5).

We found that n_{eff}=25.85 for this compendium. When experiments were selected via greedy search to maximize n_{eff}, we observed a peak of n_{eff}=26.79 using 40 experiments (Supplementary Figure S8(A)). The facts that the peak is attained using almost the entire data set, and that peak n_{eff} is not much greater than overall n_{eff}, demonstrate that in this setting, where experiments were carefully selected to cover a diverse range of conditions, most experiments are contributing positively to the effective sample size. This is in contrast to the larger M3D compendium, where experiments were taken from multiple labs, often looking at small variations of a condition of interest (and/or at similar conditions across labs), where we observed a sizable peak in n_{eff} using less than one third of the data (see main text Figure 4).

We then applied our TRNI and FDR analyses to this compendium. Using the entire compendium for correlation-based TRNI, we found that AUC10=2.6e-03. This is notably poorer performance in TRNI than we observed for the M3D compendium (AUC10=0.029). For example, at 30% precision, we observed 5% sensitivity for the M3D compendium (corresponding to 227 correctly inferred edges from RegulonDB), whereas we observed 0.5% sensitivity for the Zare compendium (25 edges from RegulonDB). Furthermore, we note that using 40 experiments at random from the M3D compendium resulted in AUC10 $>$ 0.017 (see main text Figure 7). The poorer performance of the Zare compendium relative to the M3D compendium is consistent with results in Zare *et al.* [11] when they applied their method to their data set and an earlier iteration of the M3D data set. The

poorer TRNI performance of the Zare data set also highlights the fact that the relative magnitude of our estimate of n_{eff} for different compendia is not predictive of the relative performance of those compendia in a given task (e.g, TRNI), as well as the fact that a compendium composed of mostly informative experiments (as measured by n_{eff}) does not indicate how informative that data set will be to a given task.

We evaluated the performance of subsets of the compendium in TRNI (Supplementary Figure S8(B)). We found that subsets did not significantly outperform the full data set, which is consistent with what we observed in Supplementary Figure S8(A), where nearly the entire data set was used to attain maximal n_{eff}. In this case, where nearly all experiments contribute positively to n_{eff}, they generally contribute positively to TRNI performance. There are subset sizes at which the n_{eff}-based and cluster-based subsets appear to outperform random subsets (subset size s=24-32), but performance is not maintained as the subset size grows, and standard deviations are large (related to the generally small AUC10 values).

We used our estimate of n_{eff} to compute n_{eff}-adjusted p-values for edges inferred via correlation-based TRNI using the Zare compendium and evaluated nominal FDR vs. empirical (RegulonDB-based) FDR (Supplementary Figure S8(C)). We observed that, while using n_{eff} yielded slight improvement over using n (green curve vs. blue curve), FDR control was generally inaccurate. We also found that the `fdrtool` methods (discussed above in Section 7 of the supplement) performed equally poorly in this analysis (red curve, representative best result from `fdrtool` methods). This is likely attributable to the overall lesser degree of informativeness of this dataset for TRNI (witness, for example, that there is a 10-fold decrease in AUC10 relative to the M3D compendium performance), which, as mentioned above, is consistent with poorer performance of the Zare data set compared to the M3D compendium observed in [11]. This in turn may perhaps be due to differences in microarray platform, as the Zare data are cDNA microarrays, while the M3D compendium is composed of Affymetrix microarrays.

Sangurdekar *et al.* used part of the Zare compendium in [8]. As part of their work, they produce a median activity score per experiment that captures how often pre-determined gene classes (sets of related genes) are active for a given experiment. This score is based on the reduction of entropy across expression values for genes in a given class for a given condition, relative to the background entropy across the data set for a given gene class. As a reduction in entropy implies increased correlation, we would expect a ranking of experiments based on this score to be inversely related to our n_{eff}-based ranking of experiments (ranked based on selection order in n_{eff} greedy search), where maximizing n_{eff} corresponds to minimizing correlation in the data set. When we compared our n_{eff}-based experiment rank to the median activity score from [8] for the 14 experiments from the Zare compendium that were included in [8], we indeed observed a relatively strong correlation between these measures (Supplementary Figure S8(D)), where top-ranked experiments for n_{eff} selection (low ranks) are generally the experiments with relatively lower median activity scores, confirming our expectation.

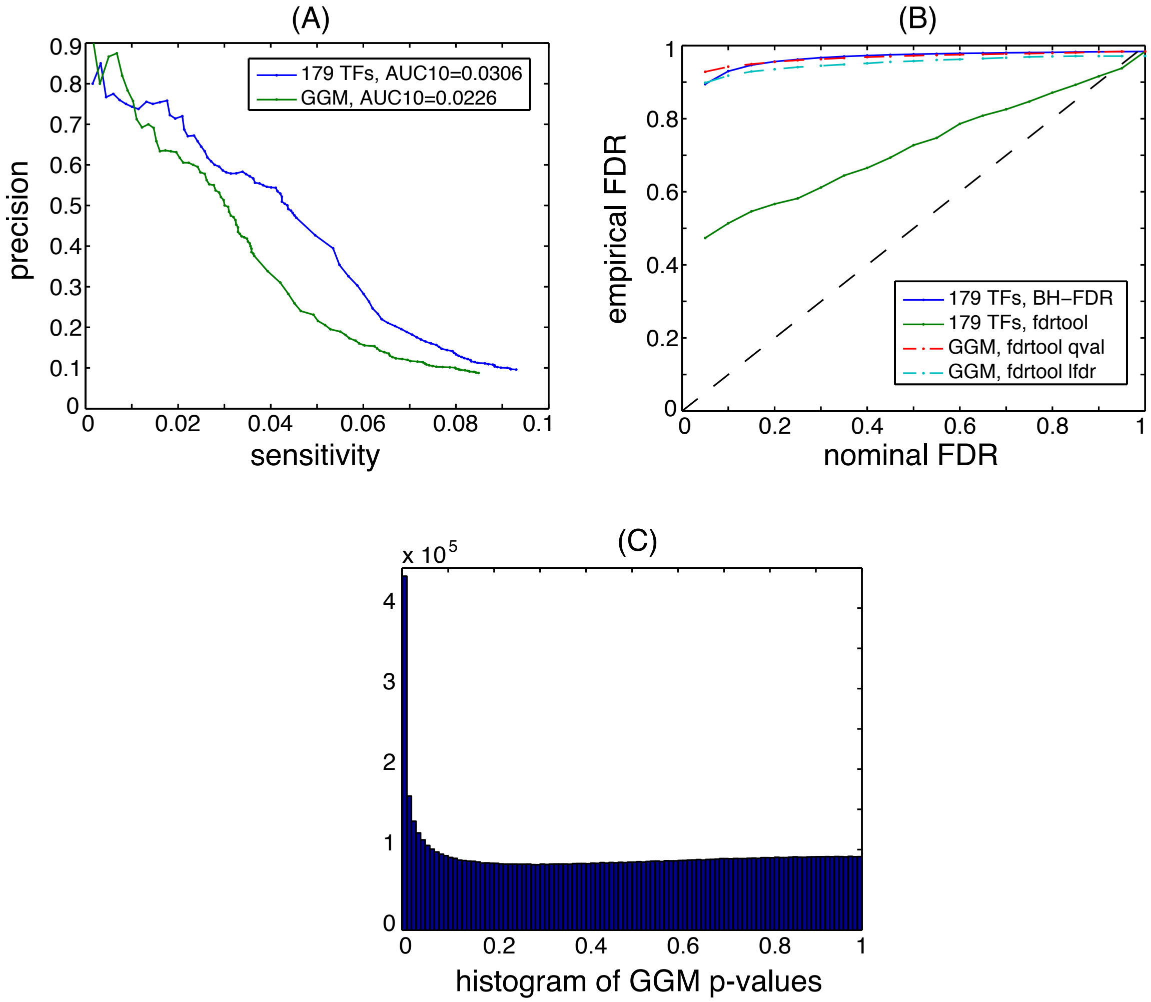

Figure 1: Supplementary Figure S1. Comparison of performance of partial correlation methods in TRNI and FDR estimation. (A) Precision vs. sensitivity for two partial correlation-based methods: 179 TF regressor set and GGM (where $k = p - 2$). (B) Empirical vs. nominal FDR for partial correlation based on 179 TF regressor set and GGM method. BH-FDR (using n) or `fdrtool` was used to control/ estimate nominal FDR for the 179 TF set results, and `fdrtool` was used to estimate tail area-based ("qval") and local ("lfdr") FDR as part of the GGM method (`fdrtool` parameter settings described in the text). Note: nominal n could not be used in the GGM case as $n < k$, yielding a negative number. (C) Histogram of p-values computed in `fdrtool` for GGM edges. (See Supplemental Text above for additional details.)

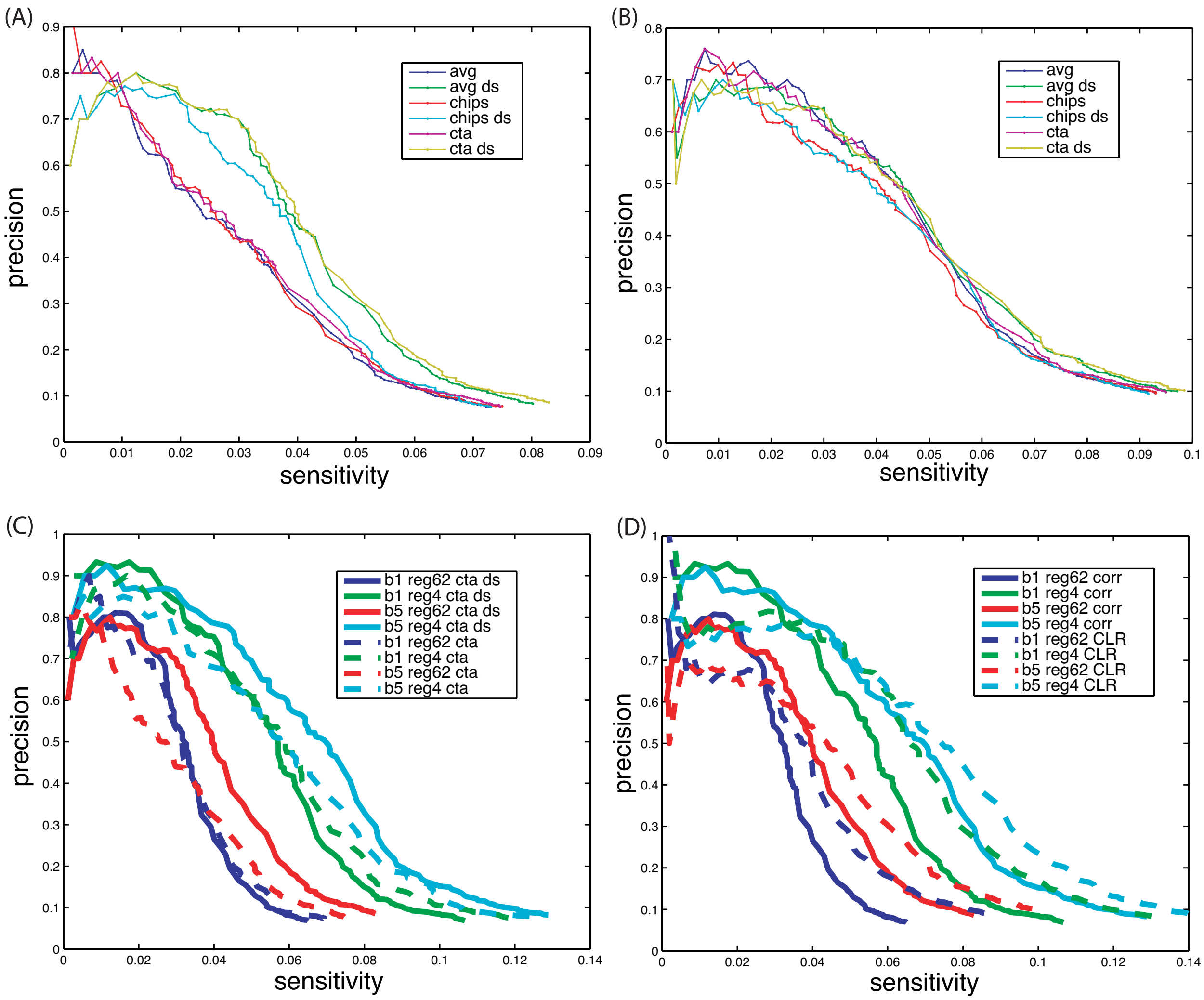

Figure 2: Supplementary Figure S2. Comparison of precision vs. sensitivity curves for various treatments of replicate experiments and standardization, and different combinations of compendium and RegulonDB versions. (A) Comparison of three treatments of replicates (including all chips, average of replicates, or closest-to-average (CTA) replicate (as described in main text)) with or without double standardization of data for correlation-based TRNI. (B) As in (A) for CLR TRNI. (C) Comparison of standardization for different combinations of compendium and RegulonDB versions for correlation-based TRNI. (D) Comparison of correlation-based or CLR TRNI for different combinations of compendium and RegulonDB versions.

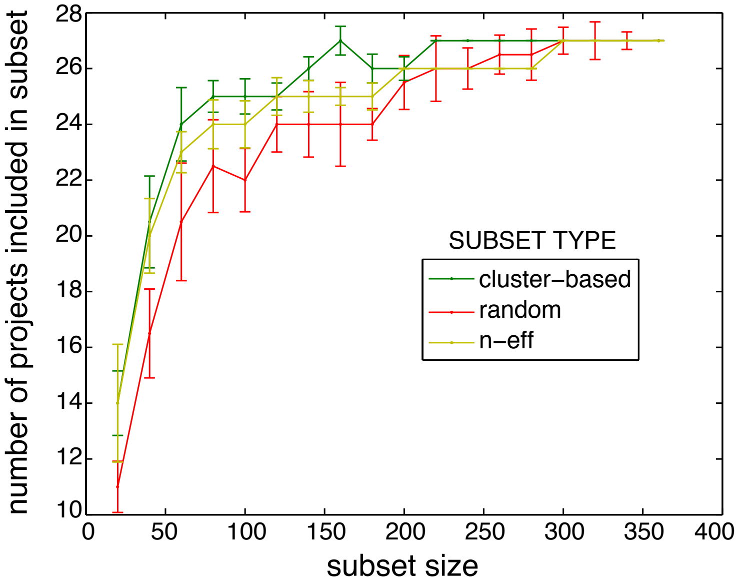

Figure 3: Supplementary Figure S3. Plots of the number of projects included in a subset vs. subset size for three types of subset selection. Each point is the median of 10 replicates and errorbars indicate +/- standard deviation.

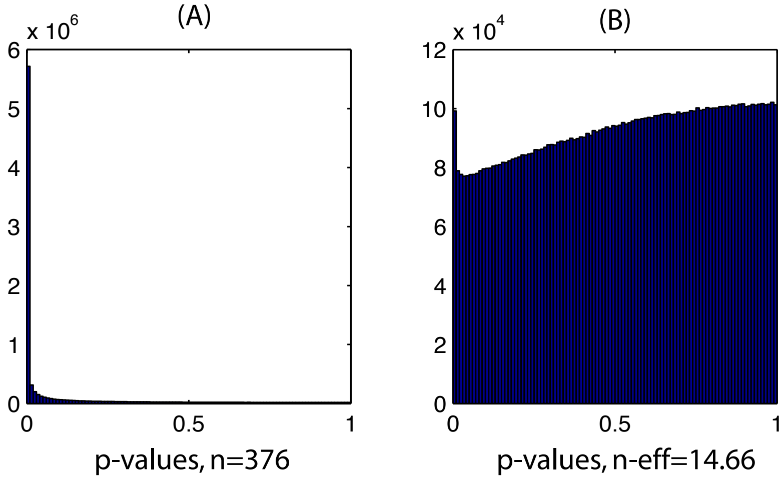

Figure 4: Supplementary Figure S4. Histograms of Fisher transformed correlation coefficient p-values for (A) n=376 and (B) n_{eff}=14.66.

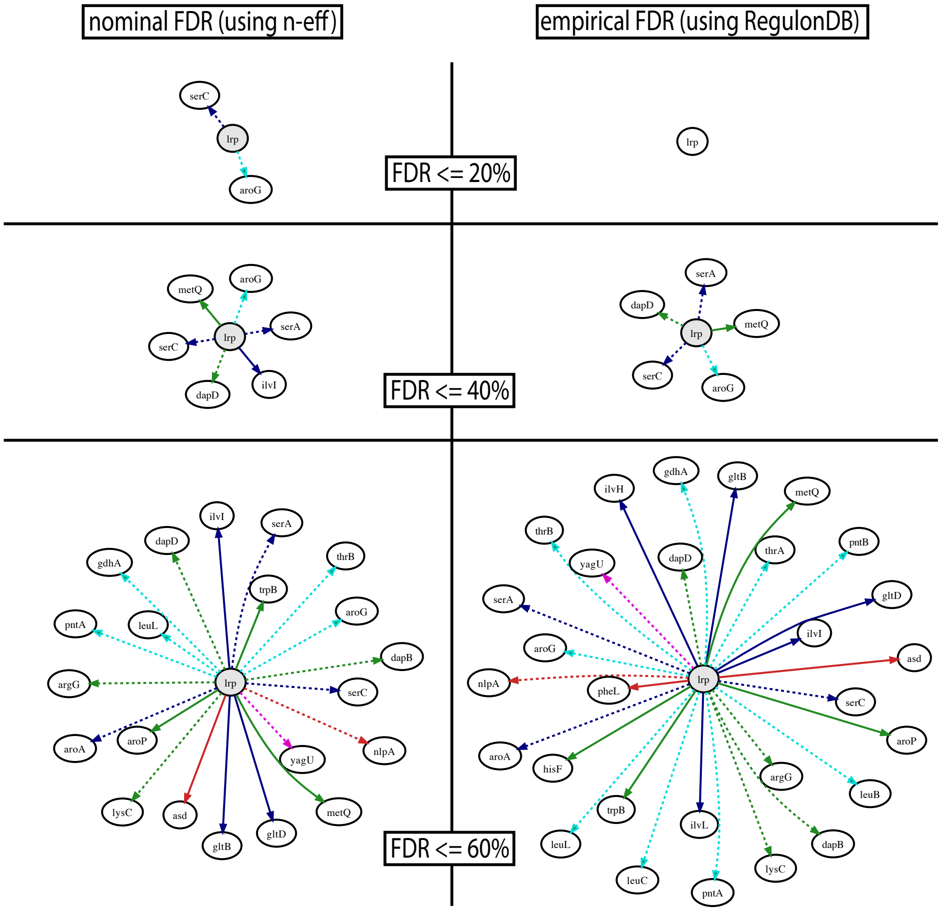

Figure 5: Supplementary Figure S5. Lrp edges for networks defined by nominal or empirical FDR estimation/ control for FDR≤20%, 40%, and 60%. Blue lines indicate known true edges according to RegulonDB; green lines indicate predicted edges for which both nodes are included in RegulonDB but no interaction is known (i.e. a false positive based on RegulonDB); and red lines indicate predicted edges for which the target gene node is not included in RegulonDB). Edges tested in ChIP-qPCR experiments carried out in [4] are indicated by dashed lines. Cyan lines indicate interactions between genes in RegulonDB that were confirmed experimentally (previously considered false positive based on RegulonDB); magenta lines indicate experimentally confirmed interactions involving a gene that isnt included in RegulonDB (i.e. a green dashed line indicates that the interaction was experimentally tested in [4] and found not to occur: an experimentally validated false positive).

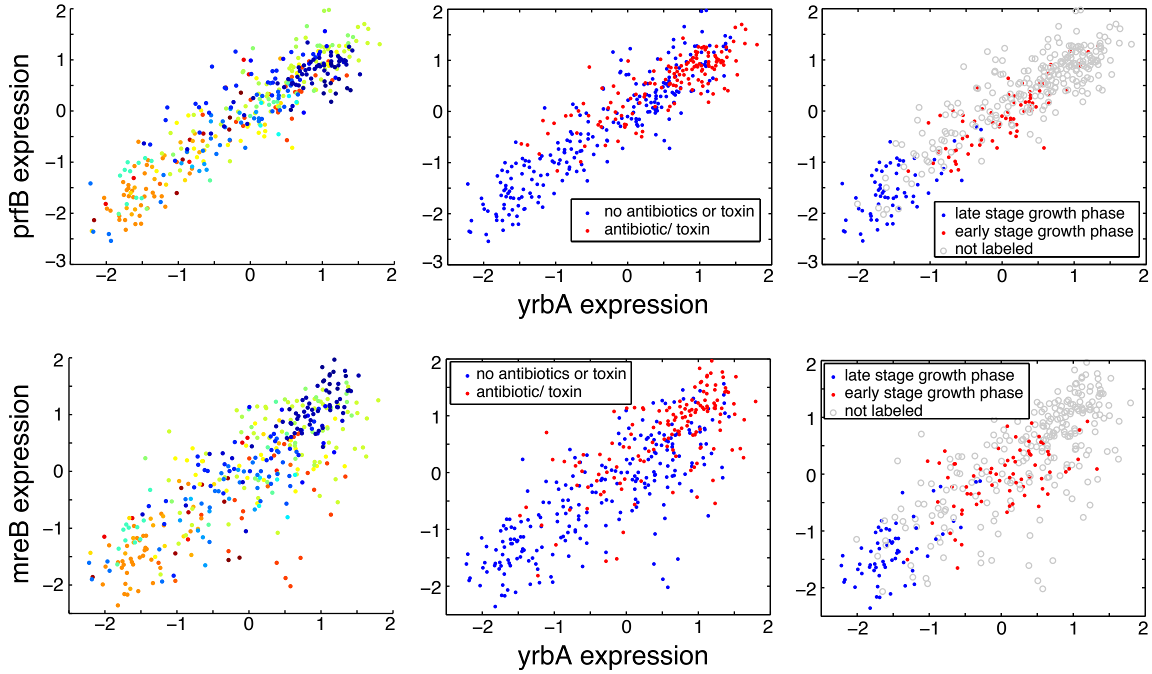

Figure 6: Supplementary Figure S6. Expression of *prfB* and *mreB* vs. putative TF gene *yrbA*. Each row displays the same plot three times, each time with points colored based on: project ID (left) (colors as in colorbar in main text Figure 1), or state of experimental condition factor specified in legend (center and right). Expression values shown are for double-standardized RMA-normalized data.

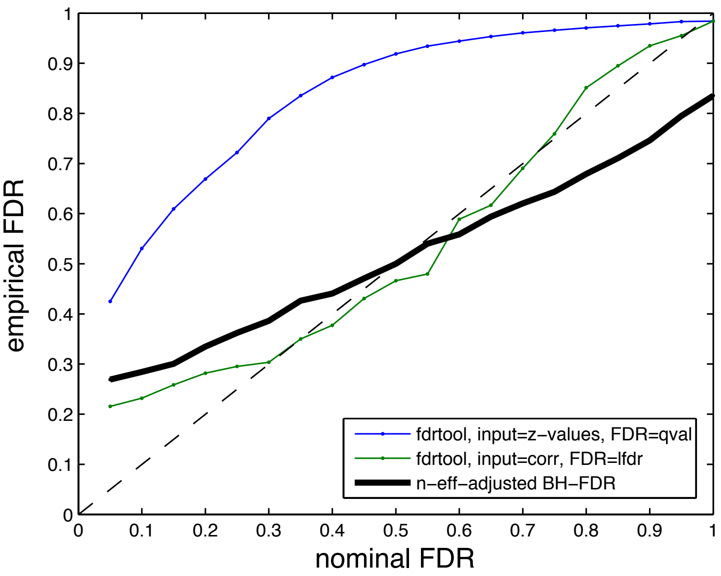

Figure 7: Supplementary Figure S7. Plots of empirical vs. nominal FDR using `fdrtool` methods, or BH-FDR adjusted by n_{eff} (bold solid curve). The legend indicates the settings of the semi-parameters in `fdrtool`: input type (correlation (corr) or z-values) and FDR type (tail area-based ("qval") or local ("lfdr") FDR). The dashed line at y=x represents the ideal case.

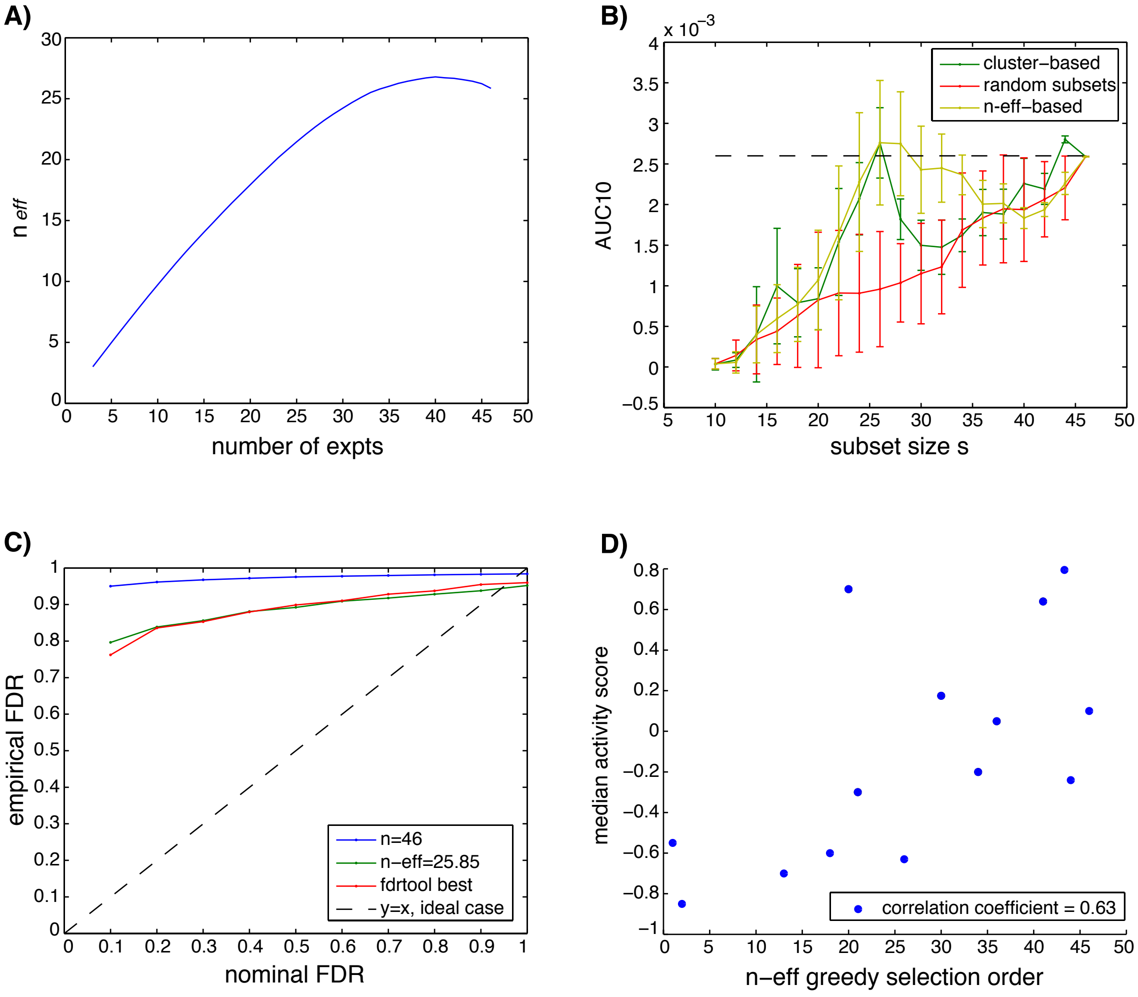

Figure 8: Supplementary Figure S8. Plots of analysis results for Zare data set [11]. Additional details provided within the supplementary text. (A) Greedy search for experiments to maximize n_{eff}. (B) Performance of subsets of the data set in TRNI. (C) Evaluation of BH-FDR control for correlation-based TRNI. (D) Comparison of experiment rank in maximizing n_{eff} to the "median activity score" of the experiment from [8].

Condition factor	Sum Sq.	d.f.	Mean Sq.	F	Prob>F
no antibiotics or ccdB	0.0599	1	0.0599	47.68	0
minimal media	0.01622	1	0.01622	12.91	0.0004
glucose	0.00402	1	0.00402	3.2	0.0745
anaerobic	0.00512	1	0.00512	4.08	0.0442
latelog, stationary, biofilm	0.23232	1	0.23232	184.89	0
Error	0.4649	370	0.00126		
Total	1	375			

Table 1: Supplementary Table S1. Multi-way ANOVA table of the effects of experimental condition factors on the first eigenvector values of corresponding experiments.

Table 2: Supplementary Table S2. Summary of networks defined based on various FDR control/ estimation methods

	FDR <= 20%											
FDR based on:	**nominal, n=376** {98%}				**nominal, n-eff** {33%}				**empirical, RegDB**			
	**RegDB subset**		**full set**		**RegDB subset**		**full set**		**RegDB subset**		**full set**	
		**TU only**		**TU only**		**TU only**		**TU only**		**TU only**		**TU only**
**corr threshold**	0.0732				0.7952				0.8941			
**# edges**	244459	127871	1065249	666490	243	101	512	308	80	34	116	53
**# genes**	1838	914	4298	2625	134	75	351	245	71	32	96	51
**# TFs**	176	170	322	311	36	32	75	68	14	13	25	22
**# hubs (>=5)**	176	170	322	311	8	7	23	19	3	2	7	3
**# self-reg (TU only)**	--	65	--	124	--	25	--	38	--	11	--	17
**# lrp edges**	1405	756	3350	2162	2	2	2	2	0	0	0	0
**# fecl edges**	1194	631	2911	1854	7	5	7	5	1	1	1	1
**# yrbA edges**	--	--	3805	2392	--	--	53	37	--	--	2	2

	FDR <= 40%											
FDR based on:	**nominal, n=376** {98%}				**nominal, n-eff** {44%}				**empirical, RegDB**			
	**RegDB subset**		**full set**		**RegDB subset**		**full set**		**RegDB subset**		**full set**	
		**TU only**		**TU only**		**TU only**		**TU only**		**TU only**		**TU only**
**corr threshold**	0.0492				0.755				0.7706			
**# edges**	269936	137893	1168411	719221	336	155	925	610	300	132	738	465
**# genes**	1838	914	4298	2625	207	121	609	417	174	101	503	350
**# TFs**	176	170	322	311	50	46	111	104	43	39	94	87
**# hubs (>=5)**	176	170	322	311	18	10	40	29	16	10	35	25
**# self-reg (TU only)**	--	65	--	124	--	29	--	48	--	26	--	42
**# lrp edges**	1542	812	3645	2315	6	6	6	6	5	5	5	5
**# fecl edges**	1397	721	3355	2101	14	8	16	10	9	6	10	7
**# yrbA edges**	--	--	3986	2488	--	--	123	76	--	--	96	58

	FDR <= 60%											
FDR based on:	**nominal, n=376** {98%}				**nominal, n-eff** {55%}				**empirical, RegDB**			
	**RegDB subset**		**full set**		**RegDB subset**		**full set**		**RegDB subset**		**full set**	
		**TU only**		**TU only**		**TU only**		**TU only**		**TU only**		**TU only**
**corr threshold**	0.0314				0.7177				0.7021			
**# edges**	289323	144864	1245814	756362	484	248	1700	1213	575	296	2206	1587
**# genes**	1838	914	4298	2625	328	189	994	673	393	219	1168	782
**# TFs**	176	170	322	311	71	67	155	148	75	71	172	165
**# hubs (>=5)**	176	170	322	311	24	15	68	53	24	16	79	67
**# self-reg (TU only)**	--	65	--	124	--	33	--	58	--	36	--	65
**# lrp edges**	1656	860	3886	2442	18	16	21	19	25	18	29	22
**# fecl edges**	1556	798	3698	2303	21	11	26	16	21	11	26	16
**# yrbA edges**	--	--	4107	2547	--	--	197	118	--	--	235	144

Table Legend

Column labels

- Row 1: indicates the nominal or empirical FDR
- Row 2: indicates how the FDR was controlled/ estimated (percent in { } indicates the RegulonDB-based empirical FDR estimate for the nominally determined FDR)
- Row 3: indicates whether only the genes present in RegulonDB (1838 genes x 176 TFs) or the full set (4298 genes x 322 TFs, where TF set included both known and putative TFs) were included in the network
- Row 4: indicates whether single genes or transcription units (TU) were used as the nodes for the network

Row labels

- corr threshold: threshold on abs(correlation coefficient) corresponding to the target FDR
- # edges: number of edges in the network
- # genes: number of genes in the network
- # TFs: number of transcription factors in the network
- # hubs (>=5): number of TFs with 5 or more edges, termed “hubs”
- # self-reg (TU only): number of “edges” corresponding to self-regulation (only happens in TU-only networks where a TF and its target(s) are in the same transcription unit (TU)
- # lrp, fecI, and yrbA edges: number of edges inferred for each of these three selected TFs

Table 3: Supplementary Table S3. Projects included in the M3D E_coli_v4_Build_5 compendium [3].

Project ID	# Expts	Short Description
1	12	over-expression of 12 different genes in presence of norfloxacin (lower concentration)
2	45	over-expression of 45 different genes in presence of norfloxacin (higher concentration)
3	10	recA knockout and luc2 over-expression at various concentrations of norfloxacin
4	8	time series experiments for norfloxacin treatment (high concentration)
5	28	time series experiments during ccdB (protein toxin) upregulation for different *E. coli* strains
6	14	deletion of 5 different genes in anaerobic and aerobic conditions
7	20	various perturbations including heat shock, acid shock, antibiotic treatment, nutrient variation, knockouts, and different growth phases
8	8	over-expression of 4 synthetic polypeptides
9	1	*E. coli* growing on succinate
10	2	expression of an amber suppressor tRNA
11	3	fnr deletion mutant during shift from aerobic to anaerobic growth conditions
12	10	IPTG-induced recombinant protein expression in high cell density cultures
13	7	different growth phases and strains under aerobic and anaerobic conditions
14	18	time series experiments over different growth phases with low or high glucose concentration
15	10	pH 5, 7, and 8.5 under aerobic and anaerobic growth conditions, and rapid acid response time series (pH 5.5)
16	58	time series experiments for various perturbations including: expression of ccdB toxin, treatment with norfloxacin, addition of iron chelator, recA knockout, *E. coli* strain (4 strains used), and combinations of these perturbations
17	27	time series experiments for 5 different antibiotics
18	8	time series experiments over different growth phases for fis deletion mutant
19	12	time series experiments over different growth phases for anaerobic and aerobic growth conditions, and one experiment in mouse cecum
20	35	various biofilm and suspension (stationary growth) cultures of 3 *E. coli* strains, including 8 knockouts, exposure to indole, time series experiments, inclusion of conjugative plasmid, and increased temperature
21	4	luxS deletion mutant with and without glucose
22	20	stringent response conditions induced by serine hydroxamate or l-valine, with perturbations including time series experiments, relA over-expression or deletion, and 2 other gene deletions
23	2	anaerobic exposure to tannins
24	4	rpoS deletion mutant during exponential and stationary growth phase
25	2	expression of hydrogenase from cyanobacterium
26	6	rpoS deletion mutant during exponential, stationary, and biofilm growth phase
27	2	expression of mutant human hsp60 in *E. coli*

Supplementary Table S4. Key experimental conditions in pairs of correlated experiments from different projects (115 pairs with FDR≤0.1).

	Expt A			Expt B	
Corr	Project ID: Name	Notable differences	Notable similarities	Notable differences	Project ID: Name
0.8679	12: har-S1-R-IPTG	strain MG1655<br>late-log growth phase<br>CAT expression (plasmid) induced by IPTG	LB miller media	strain BW25113<br>biofilm growth phase	20: BW25113-wt-7hr-biofilm
0.8562	12: har-S4-R-IPTG	strain MG1655<br>late-log growth phase<br>CAT expression (plasmid) induced by IPTG	LB miller media	strain BW25113<br>biofilm growth phase	20: BW25113-wt-7hr-biofilm
0.8406	19: MG1655-t1560-aerobic	strain MG1655<br>stationary growth phase	LB miller media	strain BW25113<br>biofilm growth phase	20: biofilm-24hr-wt-yliH-yceP
0.8393	19: MG1655-t1560-aerobic	strain MG1655<br>stationary growth phase	LB miller media	strain BW25113<br>biofilm growth phase	20: biofilm-15hr
0.8337	19: MG1655-t1560-aerobic	strain MG1655<br>stationary growth phase	LB miller media	strain BW25113<br>tnaA knockout<br>biofilm growth phase	20: biofilm-K-tnaA
0.8278	2: fklB-U-N0075	fklB overexpressed	norfloxacin 750 ng/mL<br>same experimenter<br>LB miller media<br>MG1655-yale<br>cell density=0.3	wild-type	3: WT-N0075
0.8244	19: MG1655-t1560-aerobic	strain MG1655	LB miller media<br>stationary growth phase	strain BW25113	20: suspension-24hr
0.8223	19: MG1655-t1560-aerobic	strain MG1655	LB miller media<br>stationary growth phase	strain BW25113	20: suspension-15hr
0.8077	5: lacZ-MG1063-t0	control experiment	strain MG1063<br>LB miller media<br>initial timepoints<br>same experimenter	initial timepoint	16: ccdB-chelator-MG1063-t0
0.8008	2: mcrC-U-N0075	mcrC overexpressed	norfloxacin 750 ng/mL<br>same experimenter<br>LB miller media<br>MG1655-yale<br>cell density=0.3	wild-type	3: WT-N0075
0.7979	5: lacZ-MG1063-t30	control experiment	strain MG1063<br>LB miller media<br>a control expreiment and an initial timepoint	initial timepoint	16: norfloxacin-chelator-MG1063-t0
0.7935	6: M9-K-soxS	soxS knockout<br>M9 minimal media	MG1655-derived strains<br>minimal media<br>log growth phase	wild-type<br>MOPS minimal media	26: rb-wt-exponential

Supplementary Table S4 (continued).

	Expt A			Expt B	
Corr	Project ID: Name	Notable differences	Notable similarities	Notable differences	Project ID: Name
0.7912	7: WT-MOPS-stationary3	NA	strain MG1655<br>stationary growth phase<br>MOPS minimal media	NA	26: rb-wt-stationary
0.7899	5: ccdB-MG1063-t0	NA	strain MG1063<br>LB miller media<br>initial timepoints<br>same experimenter	NA	16: ccdB-chelator-MG1063-t0
0.7893	12: har-S1-R-IPTG	strain MG1655<br>late-log growth phase<br>CAT expression (plasmid) induced by IPTG	LB miller media	strain BW25113<br>biofilm growth phase	20: biofilm-15hr-wt-yncC
0.7888	12: har-S4-R-IPTG	LB miller media<br>late-log growth phase<br>CAT expression (plasmid) induced by IPTG	strain MG1655	M9 minimal media<br>biofilm growth phase<br>R1 conjugative plasmid	20: MG1655-wt-R1drd19-24hr-biofilm
0.7887	7: WT-MOPS-glycerol	glycerol added	strain MG1655<br>MOPS minimal media<br>log growth phase	amber suppressor tRNA over-expression	10: ast-pBADsup2
0.7884	6: M9-WT	M9 minimal media	strain MG1655<br>minimal media<br>log growth phase	MOPS minimal media	26: rb-wt-exponential
0.7874	12: har-S1-R-IPTG	strain MG1655<br>late-log growth phase<br>CAT expression (plasmid) induced by IPTG	LB miller media	strain BW25113<br>yliH knockout<br>biofilm growth phase (24 hours)	20: biofilm-24hr-del-yliH
0.7866	2: b2618-U-N0075	b2618 overexpressed	norfloxacin 750 ng/mL<br>same experimenter<br>LB miller media<br>MG1655-yale<br>cell density=0.3	wild-type	3: WT-N0075
0.786	2: yebF-U-N0075	yebF overexpressed	norfloxacin 750 ng/mL<br>same experimenter<br>LB miller media<br>MG1655-yale<br>cell density=0.3	wild-type	3: WT-N0075
0.7856	7: MOPS-K-hupB	hupB knockout	MG1655-derived strains<br>MOPS minimal media<br>log growth phase	fnr knockout	11: fnr-K-fnrAerobic
0.7853	19: MG1655-t1560-aerobic	strain MG1655<br>stationary growth phase	LB miller media	strain BW25113<br>biofilm growth phase	20: biofilm-24hr

Supplementary Table S4 (continued).

	Expt A				Expt B
Corr	Project ID: Name	Notable differences	Notable similarities	Notable differences	Project ID: Name
0.785	2: mcrB-U-N0075	mcrB overexpressed	norfloxacin 750 ng/mL<br>same experimenter<br>LB miller media<br>MG1655-yale<br>cell density=0.3	wild-type	3: WT-N0075
0.783	12: har-S4-R-IPTG	strain MG1655<br>late-log growth phase<br>CAT expression (plasmid) induced by IPTG	LB miller media	strain BW25113<br>yliH knockout<br>biofilm growth phase	20: biofilm-24hr-del-yliH
0.781	2: pyrC-U-N0075	pyrC overexpressed	norfloxacin 750 ng/mL<br>same experimenter<br>LB miller media<br>MG1655-yale<br>cell density=0.3	wild-type	3: WT-N0075
0.7807	2: crcB-U-N0075	crcB overexpressed	norfloxacin 750 ng/mL<br>same experimenter<br>LB miller media<br>MG1655-yale<br>cell density=0.3	wild-type	3: WT-N0075
0.7805	6: M9-K-appY	appY knockout<br>M9 minimal media	MG1655-derived strains<br>minimal media<br>log growth phase	wild-type<br>MOPS minimal media	26: rb-wt-exponential
0.7795	2: nupC-U-N0075	nupC overexpressed	norfloxacin 750 ng/mL<br>same experimenter<br>LB miller media<br>MG1655-yale<br>cell density=0.3	wild-type	3: WT-N0075
0.779	12: har-S4-R-noIPTG	strain MG1655<br>late-log growth phase	LB miller media	strain BW25113<br>biofilm growth phase	20: BW25113-wt-7hr-biofilm
0.7777	4: T48-N10000	norfloxacin 10 ug/mL<br>48 minute timepoint	norfloxacin treatment<br>MG1655-derived strains<br>LB miller media	norfloxacin 250 ng/mL<br>30 minute timepoint	17: MG1655-norfloxacin-t30
0.7776	4: T60-N10000	norfloxacin 10 ug/mL<br>60 minute timepoint	norfloxacin treatment<br>MG1655-derived strains<br>LB miller media	norfloxacin 250 ng/mL<br>30 minute timepoint	17: MG1655-norfloxacin-t30
0.7776	5: ccdB-MG1063-t0	NA	strain MG1063<br>LB miller media<br>initial timepoints<br>same experimenter	NA	16: MG1063-uninduced-t0

Supplementary Table S4 (continued).

	Expt A			Expt B	
Corr	Project ID: Name	Notable differences	Notable similarities	Notable differences	Project ID: Name
0.7775	5: lacZ-MG1063-t0	NA	strain MG1063<br>LB miller media<br>initial timepoints<br>same experimenter	NA	16: MG1063-uninduced-t0
0.7753	5: ccdB-MG1063-t0	NA	strain MG1063<br>LB miller media<br>initial timepoints	NA	16: norfloxacin-chelator-MG1063-t0
0.7753	5: lacZ-MG1063-t0	NA	strain MG1063<br>LB miller media<br>initial timepoints	NA	16: norfloxacin-MG1063-t0
0.775	2: cpxR-U-N0075	cpxR overexpressed	norfloxacin 750 ng/mL<br>same experimenter<br>LB miller media<br>MG1655-yale<br>cell density=0.3	wild-type	3: WT-N0075
0.7732	12: har-S1-R-IPTG	LB miller media<br>late-log growth phase<br>CAT expression (plasmid) induced by IPTG	strain MG1655	M9 minimal media<br>biofilm growth phase<br>R1 conjugative plasmid	20: MG1655-wt-R1drd19-24hr-biofilm
0.7705	6: M9-K-arcA	arcA knockout<br>M9 minimal media	MG1655-derived strains<br>minimal media<br>log growth phase	wild-type<br>MOPS minimal media	26: rb-wt-exponential
0.7697	5: ccdB-MG1063-t0	NA	strain MG1063<br>LB miller media<br>initial timepoints	NA	16: norfloxacin-MG1063-t0
0.7665	2: folA-U-N0075	folA overexpressed	norfloxacin 750 ng/mL<br>same experimenter<br>LB miller media<br>MG1655-yale<br>cell density=0.3	wild-type	3: WT-N0075
0.7648	2: era-U-N0075	era overexpressed	norfloxacin 750 ng/mL<br>same experimenter<br>LB miller media<br>MG1655-yale<br>cell density=0.3	wild-type	3: WT-N0075
0.7641	2: fis-U-N0075'	fis overexpressed	norfloxacin 750 ng/mL<br>same experimenter<br>LB miller media<br>MG1655-yale<br>cell density=0.3	wild-type	3: WT-N0075

Supplementary Table S4 (continued).

	Expt A				Expt B
Corr	Project ID: Name	Notable differences	Notable similarities	Notable differences	Project ID: Name
0.7638	7: WT-MOPS-glucose	glucose added	MG1655-derived strains<br>MOPS minimal media<br>log growth phase	fnr knockout	11: fnr-K-fnrAerobic
0.7636	2: yoeB-U-N0075	norfloxacin<br>yoeB overexpressed	LB miller media<br>MG1655-derived strains	spectinomycin treatment	17: sg-spect-t30
0.7634	12: har-S4-R-IPTG	strain MG1655<br>late-log growth phase<br>CAT expression (plasmid) induced by IPTG	LB miller media	strain BW25113<br>biofilm growth phase	20: biofilm-15hr-wt-yncC
0.7626	2: galF-U-N0075	galF overexpressed	norfloxacin 750 ng/mL<br>same experimenter<br>LB miller media<br>MG1655-yale<br>cell density=0.3	wild-type	3: WT-N0075
0.7621	5: lacZ-MG1063-t60	NA	strain MG1063<br>LB miller media<br>control experiments<br>same experimenter<br>60 minute timepoint	NA	16: MG1063-uninduced-t60
0.762	2: bcp-U-N0075	bcp overexpressed	norfloxacin 750 ng/mL<br>same experimenter<br>LB miller media<br>MG1655-yale<br>cell density=0.3	wild-type	3: WT-N0075
0.7595	7: MOPS-K-dps-stationary2	dps knockout<br>MOPS minimal media	MG1655-derived strains<br>stationary growth phase	LB miller media	19: MG1655-t1560-aerobic
0.7576	2: menB-U-N0075	menB overexpressed	norfloxacin 750 ng/mL<br>same experimenter<br>LB miller media<br>MG1655-yale<br>cell density=0.3	wild-type	3: WT-N0075
0.7576	7: MOPS-K-dps-stationary	dps knockout<br>strain MG1655	MOPS minimal media<br>stationary growth phase	strain BW30270	13: cybr-O-stat
0.7563	5: ccdB-MG1063-t30	ccdB toxin	strain MG1063<br>LB miller media<br>ccdB toxin and norfloxacin have similar mode of action	norfloxacin	16: norfloxacin-MG1063-t30
0.7545	7: WT-MOPS-glycerol	glycerol added	strain MG1655<br>MOPS minimal media<br>log growth phase	succinate added	9: carbonSourceForaging

Supplementary Table S4 (continued).

	Expt A			Expt B	
Corr	Project ID: Name	Notable differences	Notable similarities	Notable differences	Project ID: Name
0.7531	12: har-S0-noIPTG	strain MG1655<br>late-log growth phase	LB miller media	strain BW25113<br>biofilm growth phase	20: BW25113-wt-7hr-biofilm
0.7519	12: har-S4-R-IPTG	strain MG1655<br>late-log growth phase<br>CAT expression (plasmid) induced by IPTG	LB miller media	strain ATCC25404<br>biofilm growth phase<br>R1 conjugative plasmid	20: ATCC25404-wt-R1drd19-24hr-biofilm
0.7518	7: MOPS-K-dps-stationary2	dps knockout	MG1655-derived strains<br>stationary growth phase<br>MOPS minimal media	wild-type	26: rb-wt-stationary
0.7516	7: WT-MOPS-stationary3	MOPS minimal media	strain MG1655<br>stationary growth phase	LB miller media	19: MG1655-t1560-aerobic
0.7515	5: lacZ-MG1063-t30	NA	strain MG1063<br>LB miller media<br>control experiments<br>same experimenter	NA	16: MG1063-uninduced-t60
0.7512	17: MG1063-uninduced-t180	strain MG1063<br>180 minute timepoint	LB miller media	strain BW25113<br>trpE knockout<br>biofilm growth phase	20: biofilm-K-trpE
0.7506	12: har-S1-R-IPTG	strain MG1655<br>late-log growth phase<br>CAT expression (plasmid) induced by IPTG	LB miller media	strain ATCC25404<br>biofilm growth phase<br>R1 conjugative plasmid	20: ATCC25404-wt-R1drd19-24hr-biofilm
0.7498	17: sg-spect-t30	spectinomycin treatment<br>LB miller media<br>log growth phase	MG1655-derived strains	relA knockout<br>MOPS minimal media<br>serine hydroxymate treatment	22: str-str-SH-K-relA-120m
0.7472	2: minD-U-N0075	minD overexpressed	norfloxacin 750 ng/mL<br>same experimenter<br>LB miller media<br>MG1655-yale<br>cell density=0.3	wild-type	3: WT-N0075
0.7471	5: lacZ-MG1063-t30	NA	strain MG1063<br>LB miller media<br>control experiments<br>same experimenter	NA	16: MG1063-uninduced-t0
0.7467	19: MG1655-t1560-aerobic	LB miller media	strain MG1655<br>stationary growth phase	MOPS minimal media	26: rb-wt-stationary
0.7463	12: har-S1-noIPTG	strain MG1655<br>late-log growth phase	LB miller media	strain BW25113<br>biofilm growth phase	20: BW25113-wt-7hr-biofilm

Supplementary Table S4 (continued).

	Expt A				Expt B
Corr	Project ID: Name	Notable differences	Notable similarities	Notable differences	Project ID: Name
0.7457	5: lacZ-MG1063-t30	control experiment	strain MG1063<br>LB miller media<br>a control expreiment and an initial timepoint	initial timepoint	16: norfloxacin-MG1063-t0
0.7457	6: M9-K-oxyR	oxyR knockout	MG1655-derived strains<br>M9 minimal media<br>log growth phase	relA knockout	22: str-ctrl-K-relA-M9
0.7454	7: MOPS-K-dps-stationary2	MOPS minimal media<br>strain MG1655<br>dps knockout	stationary growth phase	LB miller media<br>strain BW25113	20: suspension-24hr
0.7453	7: WT-MOPS-acidShock	acid shock (pH 2 for 10 minutes)	strain MG1655<br>MOPS minimal media<br>log growth phase	succinate added	9: carbonSourceForaging
0.745	6: M9-K-fnr	fnr knockout	MG1655-derived strains<br>M9 minimal media<br>log growth phase	relA knockout	22: str-ctrl-K-relA-M9
0.7448	7: WT-MOPS-glycerol	glycerol added	MG1655-derived strains<br>MOPS minimal media<br>log growth phase	fnr knockout	11: fnr-K-fnrAerobic
0.7444	5: lacZ-MG1063-t30	control experiment	strain MG1063<br>LB miller media<br>a control expreiment and an initial timepoint<br>same experimenter	initial timepoint	16: ccdB-chelator-MG1063-t0
0.7441	4: T60-N10000	norfloxacin 10 ug/mL<br>60 minute timepoint	norfloxacin treatment<br>MG1655-derived strains<br>LB miller media	norfloxacin 250 ng/mL<br>120 minute timepoint	17: MG1655-norfloxacin-t120
0.7435	7: WT-MOPS-acidShock	acid shock (pH 2 for 10 minutes)	MG1655-derived strains<br>MOPS minimal media<br>log growth phase	fnr knockout	11: fnr-K-fnrAerobic
0.7433	12: har-S4-R-noIPTG	strain MG1655<br>late-log growth phase	LB miller media	strain BW25113<br>biofilm growth phase	20: biofilm-15hr-wt-yncC
0.7425	12: har-S0-noIPTG	strain MG1655<br>late-log growth phase	LB miller media	strain BW25113<br>biofilm growth phase	20: biofilm-15hr-wt-yncC
0.7419	4: T36-N10000	norfloxacin 10 ug/mL<br>36 minute timepoint	norfloxacin treatment<br>MG1655-derived strains<br>LB miller media	norfloxacin 250 ng/mL<br>30 minute timepoint	17: MG1655-norfloxacin-t30
0.7412	17: sg-spect-t60	spectinomycin treatment<br>LB miller media<br>log growth phase	MG1655-derived strains	relA knockout<br>MOPS minimal media<br>serine hydroxymate treatment	22: str-str-SH-K-relA-120m

Supplementary Table S4 (continued).

	Expt A			Expt B	
Corr	Project ID: Name	Notable differences	Notable similarities	Notable differences	Project ID: Name
0.7403	19: MG1655-t1080-aerobic	strain MG1655<br>stationary growth phase	LB miller media	strain BW25113<br>biofilm growth phase	20: biofilm-wt-noGlucose
0.74	2: menC-U-N0075	menC overexpressed	norfloxacin 750 ng/mL<br>same experimenter<br>LB miller media<br>MG1655-yale<br>cell density=0.3	wild-type	3: WT-N0075
0.7398	12: har-S4-IPTG	strain MG1655<br>late-log growth phase	LB miller media	strain BW25113<br>yliH knockout<br>biofilm growth phase (24 hours)	20: biofilm-24hr-del-yliH
0.7395	5: lacZ-MG1063-t30	control experiment	strain MG1063<br>LB miller media<br>30 minute timepoint	norfloxacin	16: norfloxacin-MG1063-t30
0.7394	7: MOPS-K-dps-stationary2	dps knockout<br>strain MG1655<br>MOPS minimal media<br>stationary growth phase	NA	strain BW25113<br>tnaA knockout<br>LB miller media<br>biofilm growth phase (1440 minute timepoint)	20: biofilm-K-tnaA
0.7393	7: MOPS-K-dps-stationary2	dps knockout<br>strain MG1655	MOPS minimal media<br>stationary growth phase	strain BW30270	13: cybr-O-stat
0.739	6: M9-K-fnr	fnr knockout<br>M9 minimal media	MG1655-derived strains<br>minimal media<br>log growth phase	wild-type<br>MOPS minimal media	26: rb-wt-exponential
0.7389	17: MG1063-uninduced-t180	strain MG1063<br>3 hour timepoint<br>(log growth at t=0)	LB miller media	strain BW25113<br>biofilm growth phase	20: biofilm-24hr-wt-yliH-yceP
0.7362	6: M9-K-arcA-anaerobic	arcA knockout<br>anaerobic<br>M9 minimal media	MG1655-derived strains<br>minimal media<br>log growth phase	wild-type<br>aerobic<br>MOPS minimal media	26: rb-wt-exponential
0.7362	9: carbonSourceForaging	succinate added	MG1655-derived strains<br>MOPS minimal media<br>log growth phase	fnr knockout	11: fnr-K-fnrAerobic
0.7361	7: WT-MOPS-stationary3	strain MG1655<br>MOPS minimal media<br>stationary growth phase	NA	strain BW25113<br>tnaA knockout<br>LB miller media<br>biofilm growth phase (1440 minute timepoint)	20: biofilm-K-tnaA
0.736	14: ik-H2-T2.5	strain EMG2<br>glucose added<br>2.5 hour timepoint	LB miller media	strain MG1655<br>initial timepoint	16: lacZ-MG1655-t0

Supplementary Table S4 (continued).

Corr	Expt A: Project ID: Name	Expt A: Notable differences	Notable similarities	Expt B: Notable differences	Expt B: Project ID: Name
0.7355	12: har-S1-IPTG	strain MG1655<br>late-log growth phase	LB miller media	strain BW25113<br>yliH knockout<br>biofilm growth phase (24 hours)	20: biofilm-24hr-del-yliH
0.7353	6: M9-K-fnr-anaerobic	fnr knockout<br>anaerobic<br>M9 minimal media	MG1655-derived strains<br>minimal media<br>log growth phase	wild-type<br>aerobic<br>MOPS minimal media	26: rb-wt-exponential
0.7351	12: har-S4-R-IPTG	strain MG1655<br>late-log growth phase<br>CAT expression (plasmid) induced by IPTG	LB miller media	strain BW25113<br>biofilm growth phase<br>R1 conjugative plasmid	20: BW25113-R1drd19-24hr-biofilm
0.7349	2: dnaN-U-N0075	dnaN overexpressed	norfloxacin 750 ng/mL<br>same experimenter<br>LB miller media<br>MG1655-yale<br>cell density=0.3	wild-type	3: WT-N0075
0.7343	7: MOPS-K-dps-stationary	dps knockout	MG1655-derived strains<br>MOPS minimal media<br>stationary growth phase	wild-type	26: rb-wt-stationary
0.7324	12: har-S1-R-IPTG	strain MG1655<br>late-log growth phase<br>CAT expression (plasmid) induced by IPTG	LB miller media	strain BW25113<br>biofilm growth phase<br>R1 conjugative plasmid	20: BW25113-R1drd19-24hr-biofilm
0.7319	12: har-S4-R-IPTG	strain MG1655<br>late-log growth phase<br>CAT expression (plasmid) induced by IPTG	LB miller media	strain ATCC25404<br>biofilm growth phase	20: ATCC25404-wt-24hr-biofilm
0.7318	12: har-S4-R-noIPTG	LB miller media<br>late-log growth phase	strain MG1655	M9 minimal media<br>biofilm growth phase<br>R1 conjugative plasmid	20: MG1655-wt-R1drd19-24hr-biofilm
0.7311	2: sbcB-U-N0075	sbcB overexpressed	norfloxacin 750 ng/mL<br>same experimenter<br>LB miller media<br>MG1655-yale<br>cell density=0.3	wild-type	3: WT-N0075
0.731	7: WT-MOPS-stationary3	strain MG1655	MOPS minimal media<br>stationary growth phase	strain BW30270	13: cybr-O-stat
0.7307	12: har-S4-R-noIPTG	strain MG1655<br>late-log growth phase	LB miller media	strain BW25113<br>yliH knockout<br>biofilm growth phase (24 hours)	20: biofilm-24hr-del-yliH

Supplementary Table S4 (continued).

Corr	Expt A: Project ID: Name	Expt A: Notable differences	Notable similarities	Expt B: Notable differences	Expt B: Project ID: Name
0.7305	12: har-S1-noIPTG	strain MG1655<br>late-log growth phase	LB miller media	strain BW25113<br>yliH knockout<br>biofilm growth phase (24 hours)	20: biofilm-24hr-del-yliH
0.7299	6: M9-K-arcAfnr	arcA and fnr knockout	MG1655-derived strains<br>M9 minimal media<br>log growth phase	relA knockout	22: str-ctrl-K-relA-M9
0.7296	12: har-S1-IPTG	strain MG1655<br>late-log growth phase	LB miller media	strain BW25113<br>biofilm growth phase	20: BW25113-wt-7hr-biofilm
0.7293	17: sg-spect-t120	spectinomycin treatment<br>LB miller media<br>log growth phase	MG1655-derived strains	relA knockout<br>MOPS minimal media<br>serine hydroxymate treatment	22: str-str-SH-K-relA-120m
0.7286	7: MOPS-K-dps-stationary2	dps knockout<br>strain MG1655<br>MOPS minimal media<br>stationary growth phase	NA	strain BW25113<br>LB miller media<br>biofilm growth phase (24 hours)	20: biofilm-24hr-wt-yliH-yceP
0.7285	7: WT-MOPS-stationary3	MOPS minimal media<br>strain MG1655	stationary growth phase	LB miller media<br>strain BW25113	20: suspension-24hr
0.7272	7: MOPS-K-dps	dps knockout	MG1655-derived strains<br>MOPS minimal media<br>log growth phase	fnr knockout	11: fnr-K-fnrAerobic
0.7272	20: biofilm-15hr	LB miller media<br>strain BW25113<br>biofilm growth phase	NA	MOPS minimal media<br>strain MG1655<br>stationary growth phase	26: rb-wt-stationary
0.7252	13: cybr-O-stat	strain BW30270<br>MOPS minimal media	stationary growth phase	strain MG1655<br>LB miller media	19: MG1655-t1560-aerobic
0.7251	20: suspension-24hr	strain BW25113	LB miller media<br>stationary growth phase	strain MG1655	24: wt-rpoS-stat
0.7244	12: har-S4-IPTG	strain MG1655<br>late-log growth phase	LB miller media	strain BW25113<br>biofilm growth phase	20: BW25113-wt-7hr-biofilm
0.7242	2: gcvR-U-N0075	gcvR overexpressed	norfloxacin 750 ng/mL<br>same experimenter<br>LB miller media<br>MG1655-yale<br>cell density=0.3	wild-type	3: WT-N0075
0.7239	14: ik-L2-T3	strain EMG2<br>glucose added<br>3 hour timepoint	LB miller media	strain MG1655<br>initial timepoint	16: lacZ-MG1655-t0

Table 5: Supplementary Table S5. Experimental conditions in the Zare *et al.* data set [11].

Experiment ID	Experimental condition
1	WildTypeGrowth
2	Ampicillin
3	Anaerobic
4	CalciumChloride
5	gyrAparCNorfloxacin
6	IndoleAcrylate
7	Norfloxacin
8	Novobiocin
9	RecoveryFromStationary
10	SodiumAzide
11	UV-Mean
12	Gamm-Mean
13	damage-relaxation-Mean
14	relaxation-Mean
15	Trp-starvation-Mean
16	Minimal-Rich
17	Kan-Translation-Inhibition-Mean
18	LB-stationary-Mean
19	dnaC-upshiftMedian
20	SaltStress-wt-Mean-rat
21	SaltStress-rpoS-mean-rat
22	delta-topA
23	Alanine
24	Arginine
25	Cysteine
26	Glutamate
27	Glutamine
28	Histidine
29	Lysine
30	Methionine
31	Serine
32	Threonine
33	Aspartate
34	Glycine
35	Isoleucine
36	Phenylalanine
37	Proline
38	Tryptophan
39	Valine
40	Adenosine
41	Guanosine
42	Thymidine
43	Uridine
44	Cytidine
45	TLD
46	deoC-Thymidine

References

[1] A J Butte and I S Kohane. Unsupervised knowledge discovery in medical databases using relevance networks. *Proc AMIA Symp*, pages 711–715, 1999.

[2] Bradley Efron. Are a set of microarrays independent of each other? *Annals of Applied Statistics*, 3(3):922–942, 2009.

[3] Jeremiah J Faith, Michael E Driscoll, Vincent A Fusaro, Elissa J Cosgrove, Boris Hayete, Frank S Juhn, Stephen J Schneider, and Timothy S Gardner. Many microbe microarrays database: uniformly normalized affymetrix compendia with structured experimental metadata. *Nucleic Acids Res*, 36(Database issue):D866–70, Jan 2008.

[4] Jeremiah J Faith, Boris Hayete, Joshua T Thaden, Ilaria Mogno, Jamey Wierzbowski, Guillaume Cottarel, Simon Kasif, James J Collins, and Timothy S Gardner. Large-scale mapping and validation of escherichia coli transcriptional regulation from a compendium of expression profiles. *PLoS Biol*, 5(1):e8, Jan 2007.

[5] Socorro Gama-Castro, Veronica Jimenez-Jacinto, Martin Peralta-Gil, Alberto Santos-Zavaleta, Monica I Penaloza-Spinola, Bruno Contreras-Moreira, Juan Segura-Salazar, Luis Muniz-Rascado, Irma Martinez-Flores, Heladia Salgado, Cesar Bonavides-Martinez, Cei Abreu-Goodger, Carlos Rodriguez-Penagos, Juan Miranda-Rios, Enrique Morett, Enrique Merino, Araceli M Huerta, Luis Trevino-Quintanilla, and Julio Collado-Vides. Regulondb (version 6.0): gene regulation model of escherichia coli k-12 beyond transcription, active (experimental) annotated promoters and textpresso navigation. *Nucleic Acids Res*, 36(Database issue):D120–4, Jan 2008.

[6] Rafael A Irizarry, Benjamin M Bolstad, Francois Collin, Leslie M Cope, Bridget Hobbs, and Terence P Speed. Summaries of affymetrix genechip probe level data. *Nucleic Acids Res*, 31(4):e15, Feb 2003.

[7] Eric D Kolaczyk. *Statistical Analysis of Network Data: Methods and Models*. Springer, New York, 2009.

[8] Dipen P Sangurdekar, Friedrich Srienc, and Arkady B Khodursky. A classification based framework for quantitative description of large-scale microarray data. *Genome Biol*, 7(4):R32, 2006.

[9] Juliane Schafer and Korbinian Strimmer. An empirical bayes approach to inferring large-scale gene association networks. *Bioinformatics*, 21(6):754–764, Mar 2005.

[10] Korbinian Strimmer. A unified approach to false discovery rate estimation. *BMC Bioinformatics*, 9:303, 2008.

[11] Hossein Zare, Dipen Sangurdekar, Poonam Srivastava, Mostafa Kaveh, and Arkady Khodursky. Reconstruction of escherichia coli transcriptional regulatory networks via regulon-based associations. *BMC Syst Biol*, 3:39, 2009.
